# Linked-Read Sequencing of Eight Falcons Reveals a Unique Genomic Architecture in Flux

**DOI:** 10.1093/gbe/evac090

**Published:** 2022-06-14

**Authors:** Justin J S Wilcox, Barbara Arca-Ruibal, Jaime Samour, Victor Mateuta, Youssef Idaghdour, Stéphane Boissinot

**Affiliations:** Center for Genomics & Systems Biology, New York University Abu Dhabi, Saadiyat Island, Abu Dhabi, United Arab Emirates; Al Aseefa Falcon Hospital, Nad Al Sheeba, Dubai, United Arab Emirates; Wildlife Management and Falcon Medicine and Breeding Consultancy, Abu Dhabi, United Arab Emirates; Al Barhsa 1, Dubai, United Arab Emirates; Center for Genomics & Systems Biology, New York University Abu Dhabi, Saadiyat Island, Abu Dhabi, United Arab Emirates; Biology Program, New York University Abu Dhabi, Saadiyat Island, Abu Dhabi, United Arab Emirates; Center for Genomics & Systems Biology, New York University Abu Dhabi, Saadiyat Island, Abu Dhabi, United Arab Emirates; Biology Program, New York University Abu Dhabi, Saadiyat Island, Abu Dhabi, United Arab Emirates

**Keywords:** falcons, chromosomal fusions, substitution equilibrium, NUMTs, microchromosomes, biased gene conversion

## Abstract

Falcons are diverse birds of cultural and economic importance. They have undergone major lineage-specific chromosomal rearrangements, resulting in greatly reduced chromosome counts relative to other birds. Here, we use 10X Genomics linked reads to provide new high-contiguity genomes for two gyrfalcons, a saker falcon, a lanner falcon, three subspecies of peregrine falcons, and the common kestrel. Assisted by a transcriptome sequenced from 22 gyrfalcon tissues, we annotate these genomes for a variety of genomic features, estimate historical demography, and then investigate genomic equilibrium in the context of falcon-specific chromosomal rearrangements. We find that falcon genomes are not in AT–GC equilibrium with a bias in substitutions toward higher AT content; this bias is predominantly but not exclusively driven by hypermutability of CpG sites. Small indels and large structural variants were also biased toward insertions rather than deletions. Patterns of disequilibrium were linked to chromosomal rearrangements: falcons have lost GC content in regions that have fused to larger chromosomes from microchromosomes and gained GC content in regions of macrochromosomes that have translocated to microchromosomes. Inserted bases have accumulated on regions ancestrally belonging to microchromosomes, consistent with insertion-biased gene conversion. We also find an excess of interspersed repeats on regions of microchromosomes that have fused to macrochromosomes. Our results reveal that falcon genomes are in a state of flux. They further suggest that many of the key differences between microchromosomes and macrochromosomes are driven by differences in chromosome size, and indicate a clear role for recombination and biased gene conversion in determining genomic equilibrium.

**Significance**

Falcons are a particularly diverse and widespread genus of birds of particular cultural and economic importance. Falcons have also undergone recent large-scale chromosomal rearrangements to arrive at atypically low chromosome counts relative to other birds. We produced eight new high-quality falcon genomes to support general research on falcons, and we analyze these genomes to assess how chromosome loss has influenced specific aspects of genomic architecture.

## Introduction

Falcons (genus *Falco*) are among the most successful groups of birds, possessing an unparalleled geographic distribution (Gaston et al. 2005; Gill and Donsker 2019) and ∼40 species, many resulting from several radiations in the last 3 Myr (Fuchs et al. 2015). This exceptional evolutionary and ecological diversity is coupled with a long and complex history of natural and cultural association with humans (Morgan and McGovern-Hoffman 2008; Morin and Laroulandie 2012), particularly falconry, which has transformed falcons into a living human heritage (UNESCO 2016) and valuable commodities (Fleming et al. 2011). Together, these unique natural, cultural, and economic aspects of falcons make them excellent subjects for genomic studies on a range of topics that span the gap of applied and basic science (Wilcox et al. 2019).

The peculiar natural history of falcons is coupled with what is perhaps an even more peculiar genomic architecture. Through a series of interchromosomal rearrangements, falcons have lost what was hundreds of millions of years of conserved synteny in theropods (Damas et al. 2018, O’Connor et al. 2018a), and—at a chromosome count of 2*N*∼50—emerged with the lowest diploid chromosome number of any extant birds (O’Connor et al. 2018b). This is primarily the result of a loss of microchromosomes <20 MB in length (Axelsson et al. 2005). Microchromosome loss is a fixed trait in falcons, and karyotypes within falcons are minimally divergent at 2*N*∼48–52, suggesting that microchromosomes losses occurred in the common ancestor of all falcons at least 7–10 Ma. While karyotypes have not been described for the closest relatives of falcons within the subfamily Falconinae, their next closest relatives, the Caracaras (family: Falconidae; subfamily: Polyborinae) possess typical avian karyotypes of 2*N*∼80–90 (Belterman and De Boer 1990; Tagliarini et al. 2007), suggesting that the chromosomal fusions in falcons occurred more recently than this divergence, i.e., within the last 15–20 Myr (Fuchs et al. 2015). While these chromosomal fusions have been the subject of prior research (Joseph et al. 2018), their evolutionary impact at the genomic level remains largely unexplored. Microchromosomes are distinct in their composition and behavior from larger chromosomes and are characterized by higher recombination rates, higher-mutation rate, higher gene density, more CpG islands, and higher overall GC content (Axelsson et al. 2005; Ellegren 2010; Perry et al. 2020; Schield et al. 2020). Microchromosomes may also contain a higher density of structural variants (Völker et al. 2010), a lower density of repetitive elements (International Chicken Genome Sequencing Consortium 2004), and higher levels of interchromosomal chromatin (*trans-*) associations (Lui et al. 2021). Debate continues as to whether the special characteristics of microchromosomes are a product of their size or a product of their sequence motifs (O’Connor et al. 2019; Waters et al. 2021). The fusions of microchromosomes in falcons provide an opportunity to assess this. Falcons have also been reported to have several other genomic peculiarities, including extensive nuclear mitochondrial DNA segments (NUMTs) relative to most other birds (Nacer and do Amaral 2017; Liang et al. 2018), reduced TE abundance relative to other birds, and longer genic and intragenic lengths relative to other birds (Zhang et al. 2014). Further investigation is necessary to determine links between these genomic peculiarities and the unusual chromosomal configurations of falcons.

Loss and fusions of microchromosomes could have profound effects on the genomic stability of falcons (Ellegren 2010; Perry et al. 2020). Microchromosomes have been proposed as important for maintaining high-GC isochores and genomic AT–GC equilibrium (Duret et al. 2006; Ellegren 2010). Generally, birds appear to be close to AT–GC equilibrium in overall genome-wide base composition with high-GC isochores concentrated on microchromosomes (Mugal et al. 2013; Costantini and Musto 2017), and ongoing expansion of genomic heterogeneity such that high-GC regions continue to gain GC content and low GC regions continue to lose it (Webster et al. 2006). Mammals, in contrast, lost their microchomosomes in a common ancestor more than 200 Ma and are losing GC content from high-GC isochores (Duret et al. 2002; Waters et al. 2021) and moving toward greater genomic homogeneity in several lineages. The biased-gene-conversion hypothesis (BGCH) provides an explanation for these observations and proposes that high-GC isochores formed and are maintained by high recombination rates, which favor conversion and fixation of AT-to-GC substitutions. Accordingly, the BGCH suggests that high-GC isochores in birds result from high recombination rate associated with the small size of the microchromosomes on which they are concentrated, and that the loss of high-GC isochores in mammals is driven by reduced recombination rate associated with the fusion of microchromosomes into larger chromosomes. Contrasting selectionist models have also been proposed for the origin of high-GC isochores and their ongoing decline in mammals (Costantini and Musto 2017), and are based around the idea that a higher GC content confers higher chromosomal, mRNA, and protein stability at higher temperatures (Bernardi and Bernardi 1986). These selectionist models have yet to find strong empirical support, despite a wide range of thermal systems on which to test these hypotheses (Videvall 2018). In contrast, BGCH is well supported from a mechanistic perspective, but the broader links that it proposes between genomic organization and isochore composition are harder to verify, as conserved chromosomal and isochoric (co-)configurations within major amniote lineages limit opportunities to test these hypotheses.

While the BGCH suggests that the differences in patterns of AT–GC substitution between mammals and birds arise from differences in karyotype stability between these lineages (Mugal et al. 2013), the many differences between these two lineages of endotherms confound analyses and make this assertion difficult to prove. Recombination has been shown to drive GC-biased substitutions and influence the base composition of coding regions in similar ways in both mammals and birds (Rousselle et al. 2019). Biased gene conversion and CpG hypermutability are also well documented in birds (Webster et al. 2006; Mugal et al. 2015), as in other amniotes (Figuet et al. 2015), and make contrasting contributions to patterns of AT–GC equilibrium across avian genomes (Weber et al. 2014). However, due to high levels of correlation in the genomic features of birds with chromosome type (Mugal et al. 2013; Kawakami et al. 2017), the relative roles of chromosome type and similarity in sequence motifs are difficult to disentangle (Waters et al. 2021). Localized chromosomal rearrangements have, however, been shown to alter substitutional biases in a manner consistent with the BGCH in mammals and birds (Auton et al. 2012; Mugal et al. 2013). The genome-wide interchromosomal rearrangements and associated microchromosome loss in falcons provides an opportunity to study the effects of chromosome type and chromosome loss on genomic equilibrium in detail and across whole genomes. The BGCH predicts that falcons are undergoing a substitutional bias toward higher AT content, that this GC loss is concentrated on regions formerly belonging to microchromsomes and now fused onto larger chromosomes, and that GC loss is greatest in regions of high-GC content.

Here, we sequence eight falcon genomes sampled from common falcons used in falconry: two gyrfalcons, a saker falcon, a lanner falcon, three subspecies of peregrine falcons, and the common kestrel (table 1). They complement existing, albeit lower-quality assemblies for the saker falcon and peregrine falcon (Zhan et al. 2013), an already published and superior-quality assembly for the common kestrel (Cho et al. 2019), publicly available but unpublished chromosome-scale assemblies for the gyrfalcon (RefSeq accession: GCF_015220075.1) and lesser kestrel (RefSeq accession: GCF_017639655.2), and a draft genome of the prairie falcon with publicly available short reads (Doyle et al. 2018). The falcon species and subspecies chosen for this study represent diverse ecologies and are drawn from a cosmopolitan distribution. Saker falcons breed across central Eurasia and the Middle-East and take other birds and small vertebrates as prey. Gyrfalcons reside in the arctic of Eurasia and North America and take large birds as prey. Lanner falcons occur across Africa and the Mediterranean basin, as well as parts of the Middle-East and take birds and some small mammals as prey. Peregrine falcons occur on every continent except Antarctica and typically take birds as prey: the subspecies used in this paper include the Eurasian peregrine (*F. p. peregrinus*), which occurs across temperate Eurasia; the black shaheen (*F. p. peregrinator*), which occurs in southern Asia; and the Barbary falcon (*F. p. pelegrinoides*) which occurs in the Canary Islands, North Africa, and parts of the Middle-East. The common kestrel is much smaller and more distantly diverged from the other falcons included in our study. Like other kestrels, it preys primarily on small mammals (but can take birds), and occurs across Eurasia and northern Africa. Genomes are sequenced using 10X Genomics Chromium Linked-Reads sequencing, providing a first-ever genome for a lanner falcon, and greatly improved genomic assemblies for the saker falcon, and first-ever assemblies for the Barbary falcon and black shaheen subspecies of peregrines. We use the large phased scaffolds to examine compositional changes in falcon genomes and their partitioning between conserved and fused regions of the genome. We use alignments to related genomes to annotate the ancestral and current state of genomic regions, which allowed us to assess the BGCH by examining: (1) whether falcons are in AT–GC equilibrium; (2) whether microchromosomes that have fused into larger chromosomes show a particular bias toward AT substitutions; and (3) whether GC loss is in regions of higher G.

**Table 1:** Falcon Sample Information

Taxon	Common name	Abbreviation	Source (country of origin^a^)	Origins
*F. biarmicus*	Lanner Falcon	Lanner	Al Aseefa Falcon Hospital (Unknown)	Wild
*F. cherrug*	Saker Falcon	Saker	Al Aseefa Falcon Hospital (Mongolia)	Wild
*F. p. peregrinus*	Peregrine Falcon	Peregrine	Al Aseefa Falcon Hospital (Mongolia)	Wild
*F. p. pelegrinoides*	Barbary Falcon	Barbary	Al Aseefa Falcon Hospital (Unknown)	Wild
*F. p. peregrinator*	Black Shaheen	Bl. Shaheen	Dubai Falcon Center (Pakistan)	Wild
*F. rusticolus* (1)	Gyrfalcon	Gyr-1	Al Aseefa Falcon Hospital (Canada)	Captive
*F. rusticolus* (2)	Gyrfalcon	Gyr-2	Al Taf (UAE)	Captive
*F. tinnunculus*	Common Kestrel	C. Kestrel	Dubai Falcon Center (UAE)	Wild

a Country from which sampled bird originated, if known.

## Results

### Assembly Metrics

Reads were assessed for quality using FastQC. The average Pfred quality score per read was ∼36 across all genomes and we found no quality metrics of concern. We assessed for potential contamination, sequencing bias, and read bias using Kat. We found no evidence for contamination. Most genomes did, however, show a sequencing bias toward higher GC content, as indicated by a trend for higher GC content among more frequent k-mers (supplementary fig. S1, Supplementary Material online).

We sequenced 588 GB of DNA from eight falcon genomes using 10X Genomics Chromium linked-reads sequencing and assembled these using Supernova with assembly sizes ranging from 1.14 to 1.17 GB (table 2). Scaffold counts ranged from 806 in the Black shaheen to 2,041 in the common kestrel, with the corresponding scaffold *N*50 values at a high of 40.62 MB and low of 10.58 MB. Phase block *N*50 ranged from 1.04 MB for Gyr-1 to 13.9 MB for the black shaheen with a phase block *N*50 average of 6.51 MB (supplementary table S1, Supplementary Material online). Single nucleotide variant (SNVs) and indels were also phased using Longranger: over 99% of small variants were phased in all genomes with this method and phaseblock *N*50s ranged from 2.41 MB in Gyr-1 to 10.49 MB in the black shaheen with an average phase block *N*50 of 6.21 MB across all genomes. Raw coverage was greater than 50X and averaged 58X across all genomes, exceeding the 38X minimum coverage requirements and approximating the 56X optimum target recommended by 10X Genomics for assembly with Supernova. The effective coverage (i.e., with duplicates removed) ranged from 31.52 to 61.34X.

**Table 2:** Assembly metrics

Genome	Size	GC%	Scaff	Cov	*N*50	BUSCOs: SC|Dup|F |M^a^	*Falco*-UA	I-Aligned
Lanner	1.17	42.11	1591	39.22	28.94	94.4%|0.8%|1.0%|3.8%	6.154	99.99
Saker	1.16	42.14	1152	43.67	29.00	95.1%|0.4%|0.9%|3.6%	6.415	99.98
Peregrine	1.16	42.05	1136	40.72	25.88	94.8%|0.6%|1.0%|3.6%	5.461	99.99
Barbary	1.16	42.04	1296	38.50	26.06	94.3%|0.3%|1.0%|4.4%	5.646	99.98
Bl. Shaheen	1.16	42.02	806	61.34	40.62	93.8%|0.3%|1.0%|4.9%	5.974	99.99
Gyr-1	1.17	42.27	1518	45.59	20.60	95.1%|0.7%|1.0%|3.2%	6.310	100
Gyr-2	1.17	42.25	1699	43.29	17.12	95.2%|0.5%|1.2%|3.1%	6.559	99.99
C. Kestrel	1.14	41.92	2041	37.52	10.58	91.1%|0.3%|1.3%|7.3%	5.083	100

note.—Size is in GB and *N*50 is scaffold *N*50 in MB. *Falco*-UA reports the percentage of 100 kB windows that do not align to chromosome-scale falcon assemblies of the gyrfalcon and peregrine falcon. I-Aligned reports the percentage of 100 kB windows that align to chromosome-scale assemblies of other birds in Inopinaves.

Scaff, number of scaffolds. Cov, coverage after dereplication.

a Out of 8338 BUSCOs: SC = Complete Single-Copy|Dup = Complete Duplicated|F = Fragmented|M = Missing.

Genomic completeness was assessed using BUSCO to search for single-copy avian orthologs with the “aves_odb10” database containing 8338 universal single-copy avian orthologous genes (table 2). The proportion of BUSCO genes detected as single copy and complete ranged from 91.1% for the common kestrel to 95.2% for Gyr-2 with a median of 94.6% across all assemblies. The proportion of BUSCOs that could not be detected (i.e., “missing” BUSCOs) ranged from 3.1% of the database in one of the gyr falcon (Gyr-2) to 7.3% of the database in the kestrel; across all assemblies, a median of 3.7% of BUSCOs were not detected. We further assessed genomic completeness by assessing the proportion of 100 kB windows that did not align to the chromosome-scale reference assemblies for the gyrfalcon (GCF_015220075.1) and the peregrine falcon (GCA_001887755.1), and the proportion that did align to other birds within Inopinaves (the Swainson’s thrush GCF_009819885.1, kakapo GCF_004027225.2, and red-legged seriema GCA_009819825.1). The common kestrel had the lowest proportion of 100 kB windows that did not align to the reference falcon assemblies at 5.08% whereas the Gyr-2 had the highest proportion of 100 kB windows unaligned to the falcon reference assemblies at 6.56%. Across assemblies, an average of 5.95% of 100 kB windows were unaligned to the two falcon reference assemblies. More than 99.98% of all 100 kB windows aligned to other Inopinaves in all assemblies corresponding to all but 1–2 windows in the Lanner, Saker, Peregrine, Barbary, and Black Shaheen assemblies. All 100 kB windows aligned to at least one other Inopinaves genome in the Gyr-1 and Common Kestrel assemblies. Taken together, these results suggest that our assemblies are free of contamination and that they have captured some regions absent from other high-quality falcon assemblies.

The presence of scaffolds belonging to the Z and W sex chromosomes was confirmed in all falcon genomes using HMMER. Two close proximity hits to sex chromosome-specific Z (CHD-Z) and W (CHD-W) Chromosome Helicase genes (Fridolfsson and Ellegren 1999) were found on separate scaffolds in all genomes. This search also produced a hit to a short segment of a predicted CHD-2 gene on a third scaffold of all genomes. No other hits were found to our HMMER model in any genomes.

SNVs and small indel variants were called using three methods: longranger, MUMmer alignment to the common kestrel genome, and MUMmer alignment of phased diploid genomes to one another. The number of heterozygous sites among small variants was comparable across methods (supplementary fig. S2, Supplementary Material online). Heterozygous variants ranged from 623,505 SNVs/139,591 indels in Gyr-1 to 4,578,621 SNVs/627,414 indels in the common kestrel using the self-alignment; and 820,996 SNVs/155,477 indels in Gyr-1 and 4,952,485 SNVs/705,344 indels in the common kestrel using Longranger. We detect ∼86% more heterozygous SNVs in the saker falcon and ∼42% more SNVs in the peregrine than reported in previously published genomes for these species (Zhan et al. 2013). This is consistent with the expectations of improved variant calling with linked reads (Marks et al. 2019). We detect ∼4% fewer SNVs than previously reported for the common kestrel (Cho et al. 2019). The average distance between heterozygous sites varied greatly from 0.250 kB in the common kestrel to 1.75 kB in Gyr-1 (supplementary table S2, Supplementary Material online).

**Fig. 1.**
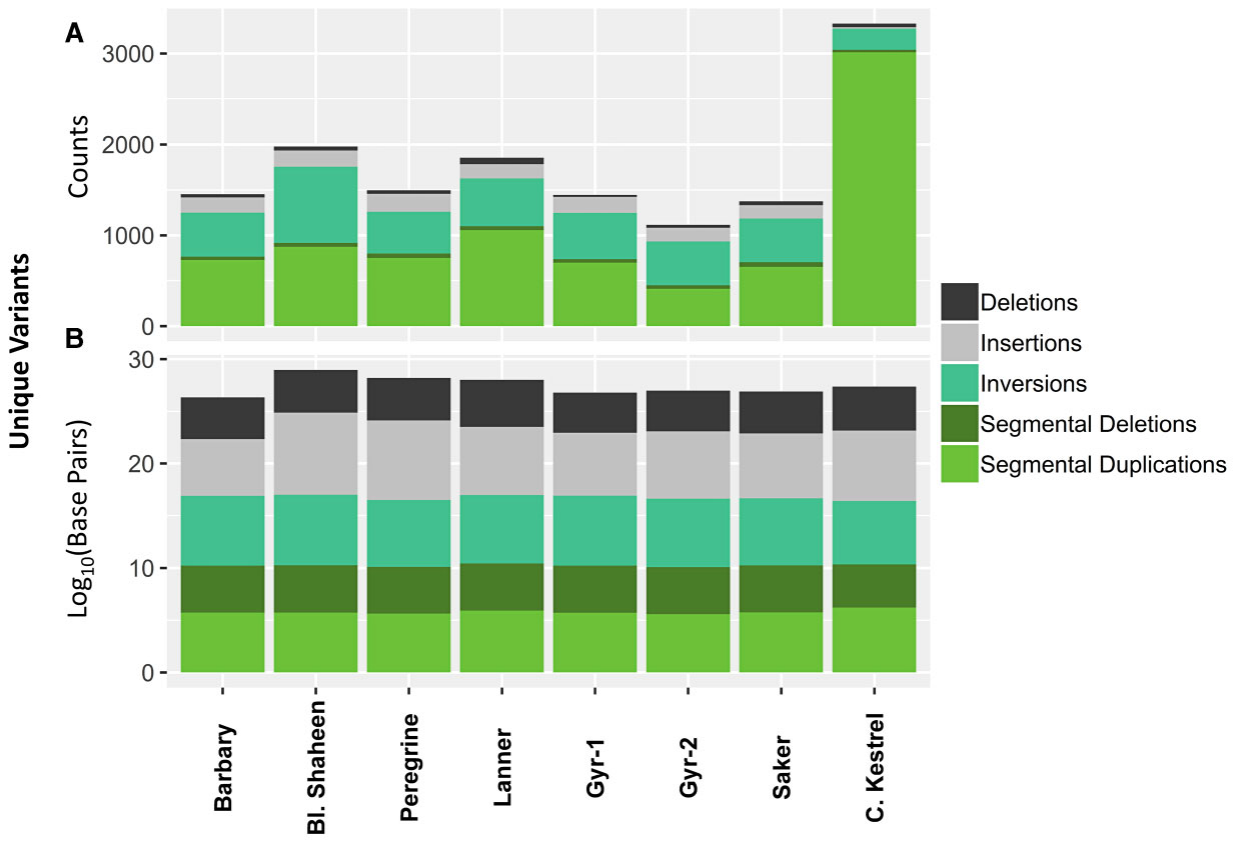
Unique structural variants within each genome based on MUMmer alignments by: (*A*) count; (*B*) log_10_(base pairs). Reported numbers are for diploid genomes and are color coded by type of structural variant. Structural variants are determined by alignment to the common kestrel outgroup. Segmental insertions and deletions are distinguished from insertions and deletions in that they represent the addition or disappearance of linked repetitive content on the same scaffold.

Structural variants were annotated from MUMmer alignments to both the kestrel genome and phased haplotypes of each individual genome. Insertions and deletions are defined relative to the common kestrel genome outgroup. Only structural variations unique (private) to each genome were considered in downstream analysis, with any structural variants overlapping any other structural variants of the same type being removed. This effectively provided a parsimony-based approach for polarizing the structural variants considered. This approach likely biases our analysis toward more recent structural variants. Structural variants private to each diploid genome varied from ∼1000 to 1500 in number (fig. 1*A*). By count, unique structural variants were dominated by inversions and segmental duplications. The numbers of insertions and deletions varied between genomes but these accounted for only a small portion of structural variants in all genomes. Unique segmental deletions were consistent in the number across genomes and ranged in count from 28 in the kestrel to 51 in the saker falcon. Unique structural variants varied in size distributions across types, and as such, counts did not reflect the differences in base pairs attributed to each type of structural variant (fig. 1*B*). Base pairs of structural variation were generally dominated by inversions, which ranged in diploid counts from 1,123,579 bp in the kestrel to 4,720,786 bp in Gyr-1. Unique inserted base pairs were extremely variable in number, and ranged from diploid counts of 1,102,918 bp in the Gyr-1, to 42,404,444 bp in the Eurasian peregrine and 78,289,714 in the Black Shaheen, with between 1.5 and 3.5 million unique inserted bases in the other falcons.

**Fig. 2.**
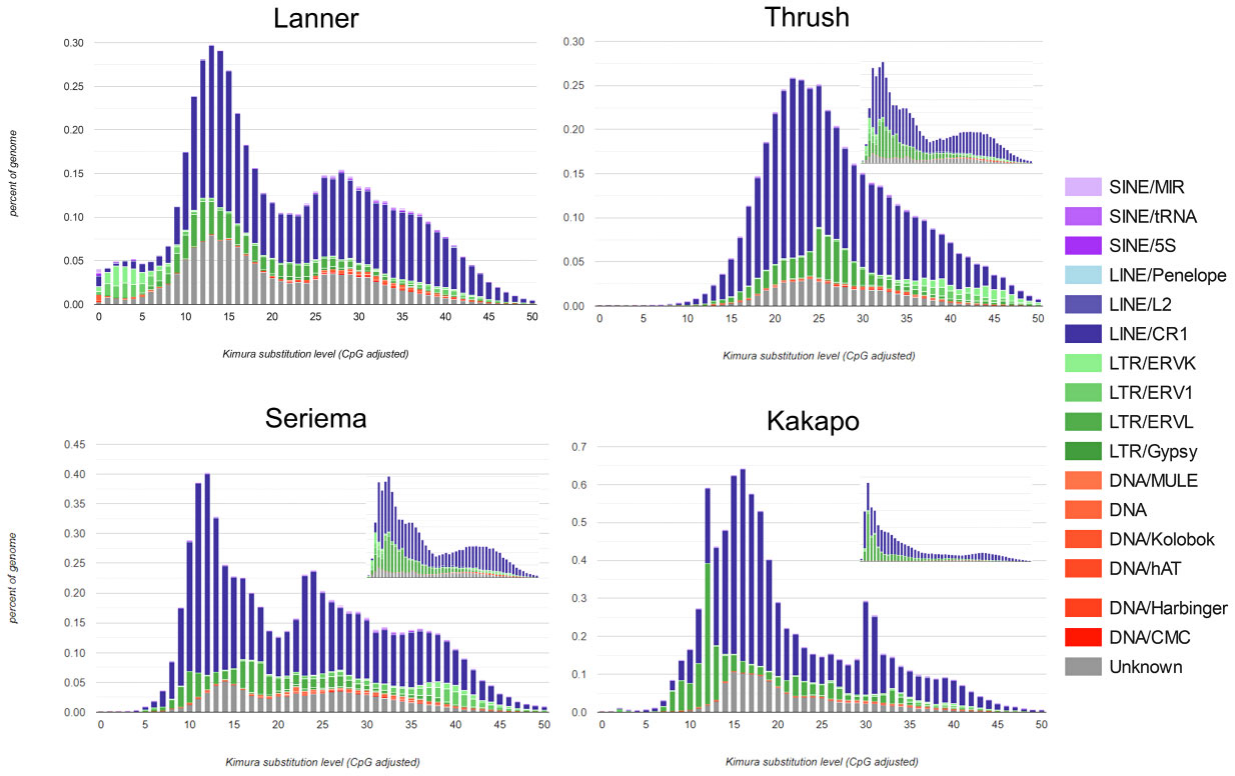
CpG adjusted divergence of repetitive elements from consensus sequences for the lanner falcon (representative of other falcons) and the three other major lineages of Inopinaves sampled from NCBI RefSeq and GenBank: the Swainson's thrush; the kakapo; and red-legged seriema. Repetitive elements are color coded by type and family. All genomes were annotated with dereplicated de novo repeat libraries compiled and dereplicated from the eight falcon genomes (large figures). The other Inopinaves genomes were also annotated using self-derived de novo libraries (small figures).

We performed a de novo repeat annotation by building a database of repeat subfamilies with RepeatModeler 2 and searching genomes for these with RepeatMasker using rmBLAST. The proportion of repeat elements annotated in each genome ranged from 6.38% in the Kestrel to 6.61% in Gyr-1. Repeat element composition was dominated by LINEs of the Chicken Repeat 1 (CR1) family with a very small number of L2 and Penelope elements detected. A small number of long terminal repeat (LTR) and “cut-and-paste” DNA elements were also detected. The divergence curves showed the same pattern for all samples with the vast majority of repeats being more than 10% divergent from consensus sequences and a particularly strong wave of amplification between 10% and 15% divergence and a somewhat older wave of amplification at 25–30% divergence for several repeat families (fig. 2). To approximate the times of these amplifications, we used the same de novo libraries from falcons to annotate repeats in birds from other clades of Eufalconimorphae (the Swainson’s Thrush and the Kakapo) and an outgroup within Australaves (the red-legged Seriema). The annotation of repeats in these other genomes suggests that both waves of amplification were already present at the time of the divergence of the Eufalconimorphae from its common ancestor with seriemas ∼60 Ma (Prum et al. 2015). Near-identical repeat landscapes between the falcons and red-legged seriema—and similar patterns in the kakapo—suggest minimal activity for the annotated repeat subfamilies in these lineages. This finding is supported by a lack of intact open reading frames (ORFs) belonging to repeats in falcons. Across all genomes, we identify 14,532–15,558 ORFs with matches to active LINE elements, but all of these had premature stop codons. We identify 10–28 ORFs belonging to LTR elements across all genomes, again all with premature stop codons. No ORFs belonging to DNA transposons were identified. Finally, we find no unique insertion variants corresponding to annotated repeats that are >300 bp in any genome. Interspersed repeats were not evenly distributed across the genome. Base pairs arising from transposons and retrotransponsons were found to be significantly enriched on 100 kB windows of the Z chromosome relative to autosomes (*P* < 1 e−6). Fusions of chromosomes could result in interstitial telomere which can be detected in the simple repeats annotated by RepeatMasker. In light of recent chromosomal fusions in falcons, we searched for simple repeats that matched either the vertebrate forward (TTAGGG) or reverse (CCCTAA) telomere sequences and that were not located on the beginning or end of a scaffold. We found between 16 and 26 simple repeats corresponding to interstitial telomeres within each diploid falcon genome.

**Fig. 3.**
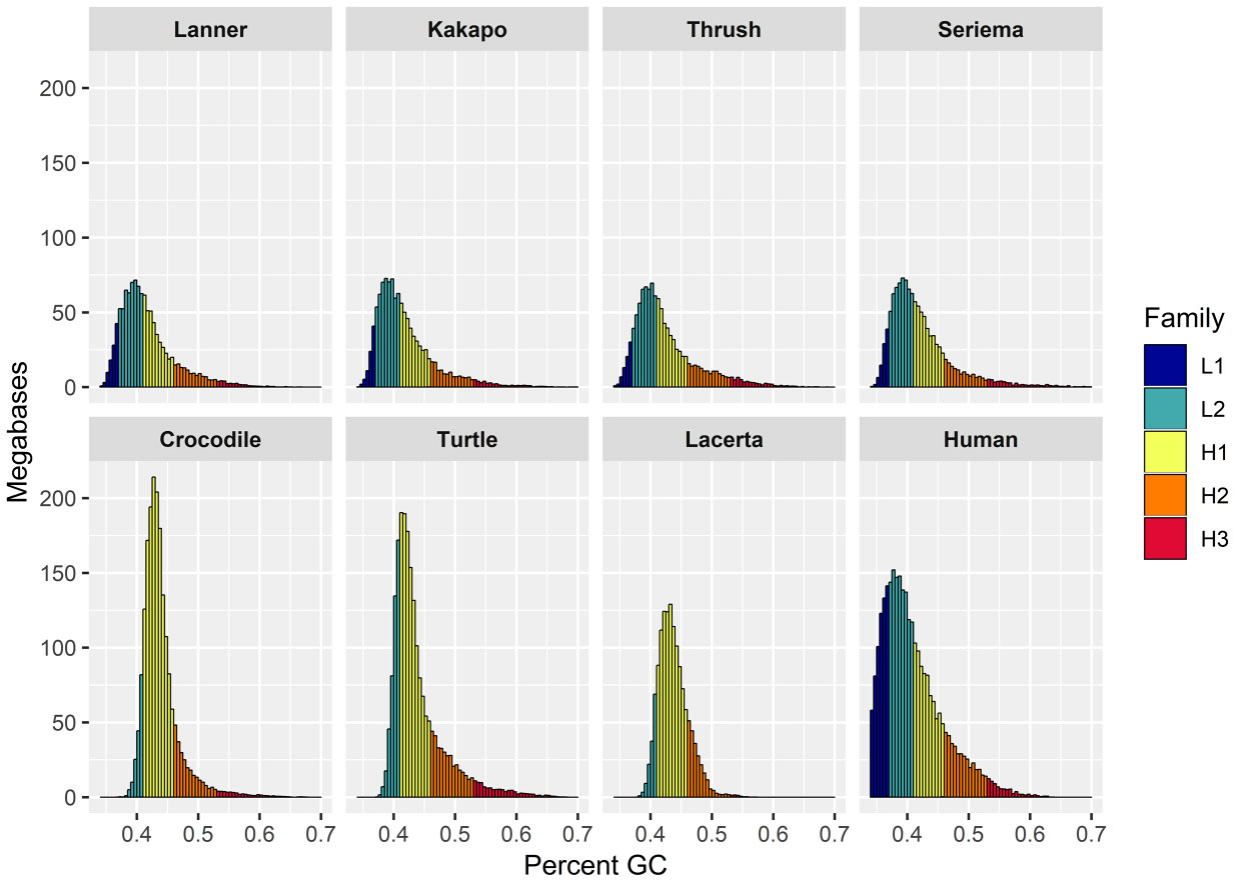
Distributions of GC content across 100 kB windows for the representative lanner falcon and other Eufalconimorphae and amniote lineages sampled from NCBI RefSeq and GenBank: the kakapo; the Swainson’s thrush; the red-legged seriema; the saltwater crocodile (*Crocodylus porosus*); the green sea turtle (*Chelonia mydas*); the sand lizard (*Lacerta agilis*); and human (*Homo sapiens*). Isochore families are delineated by color.

We annotated 100 kB (supplementary fig. S3, Supplementary Material online) and 1 MB (supplementary fig. S4, Supplementary Material online) windows to assess distributions of GC content throughout falcon genomes in the context of isochores (Costantini and Musto 2017). Falcon genomes showed similar patterns of GC distribution to one another and other birds (fig. 3). Avian genomes most closely resembled the human, but with a slight shift toward higher GC content.

### Evolutionary History

**Fig. 4.**
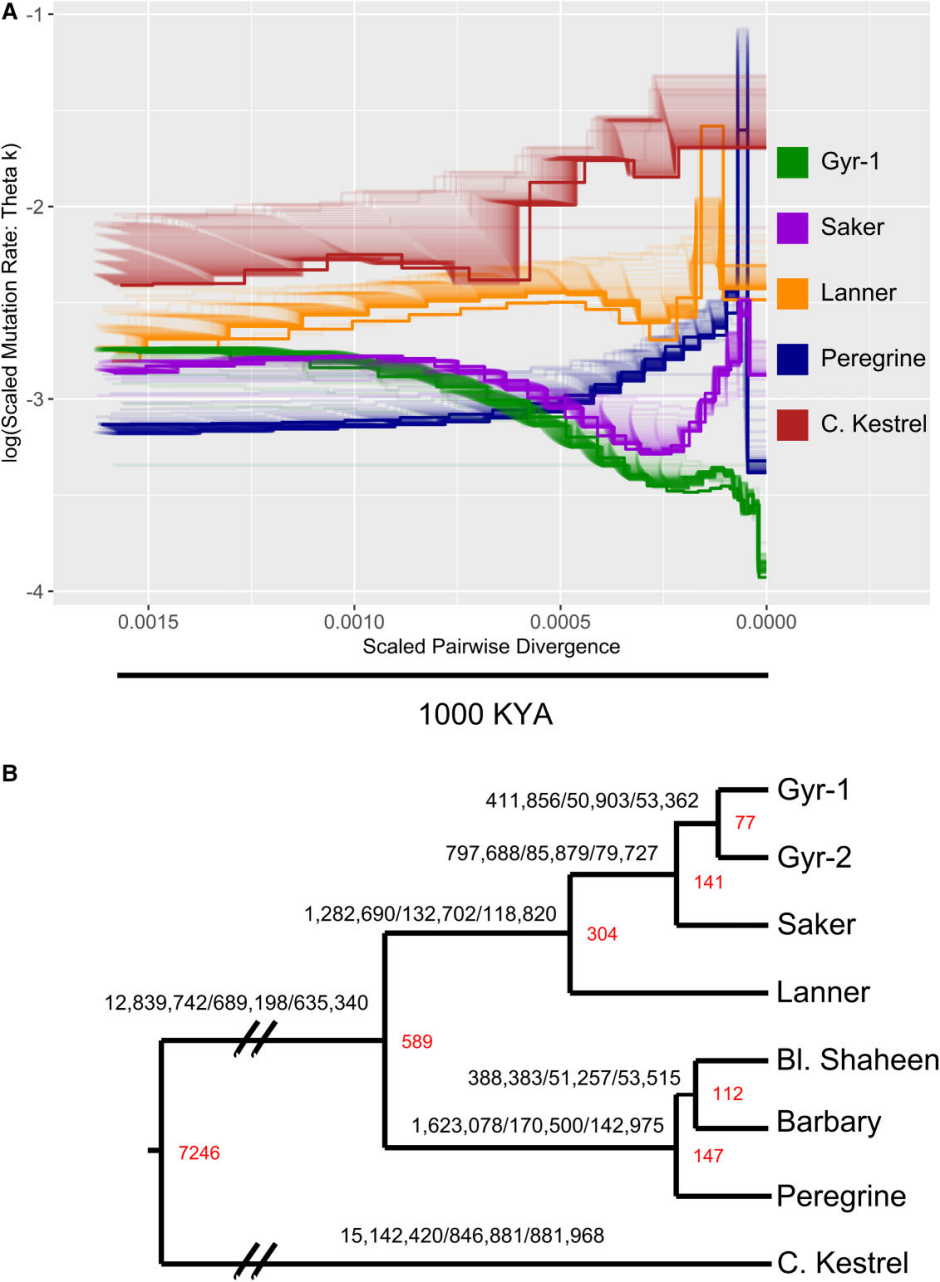
Demographic and evolutionary history of the eight falcon genomes. (*A*) PSMC: time is indicated on the *X*-axis and is represented by scaled-pairwise divergence, with higher values indicating more ancestral time points scaled to 1000 kya based on the average divergence calculated across all species. Population size is indicated on the *Y*-axis as log_10_(scaled-population-mutation rate), with higher values indicating larger effective population sizes. (*B*) Maximum-likelihood tree built using all variable SNV sites detected from alignment to the kestrel genome. The tree was dated based on divergence between the kestrels and large falcons and is shown on the same timescale as the PSMC with a truncation (//) on the long branches between the common kestrel and other falcons. Node labels (red) indicate divergence times in kya. Branch labels denote the branch-specific number of unique shared small variants <50 bp: SNVs/deletions/insertions.

A maximum-likelihood phylogeny was constructed in RAxML using all 15,426,039 variable SNV sites derived from the MUMmer alignments to the haploid kestrel genome (fig. 4*B*). The subgenus *Hierofalco* (i.e., the lanner falcon, saker falcon, and gyrfalcon) and peregrine falcons fell out into clearly defined clades. Within *Hierofalco*, the two Gyr genomes grouped together along with the saker and lanner diverging ancestrally to these. All groupings had 100% bootstrap support. The tree was scaled to the divergence of old-world kestrels and large falcons at the Tortonian–Messinian Junction, 7.246 Ma, in the late Miocene (Boev 2011). It suggests a split between the peregrine falcons and *Hierofalco* of ∼588 kya with a split between the lanner and saker falcon of ∼304 kya. The saker and gyrfalcon and the peregrine subspecies all, respectively, coalesced within the last 150 kya.

PSMCs were performed to reconstruct the demographic history of each taxon (fig. 4*A*). With the exception of the gyrfalcons, all falcons showed a historically low effective population size with a very recent increase corresponding to the last few thousand years, followed by more recent and more modest declines (supplementary fig. S5, Supplementary Material online). Gyrfalcons experienced a steady decline in population size that seems to have accelerated following their divergence from the saker falcon. The Eurasian peregrine and lanner falcon show previous expansions, ∼50 and 160 kya, respectively, followed by bottlenecks. The common kestrel began to experience a population expansion much earlier than the large falcons, at ∼200 kya.

### NUMTs Evolution

**Fig. 5.**
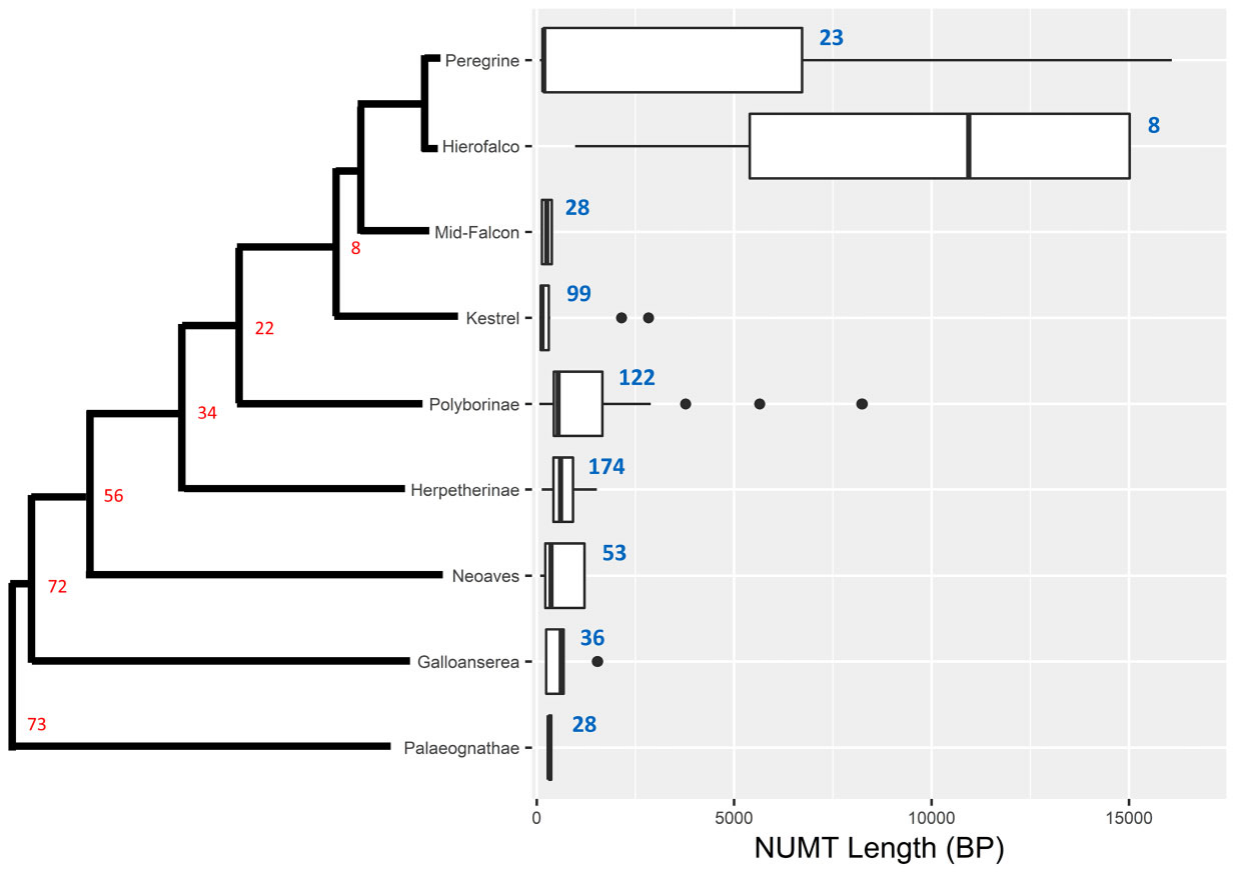
Base pair length (*x*-axis) distributions of NUMTs (labeled with count on the graph in blue) grouped by the mitochondrial genomes to which they are phylogenetically closest (*y*-axis) based on maximum-likelihood analysis. Boxplots show 1st and 3rd quartiles with the median as a bisecting line; outer lines denote up to 1.5X the interquartile range, with outliers outside this shown as circular dots. The tree to the left of the *Y*-axis shows the evolutionary history based on an ultrametric Bayesian analysis of mitogenomes. Estimated divergence times are reported from the literature (Prum et al. 2015) with nodes labels (red) in millions of years (Ma).

We annotated 900 NUMTs accounting for 992,144 bp across the eight falcon genomes of which 316 were unique to specific genomes, totaling 368,339 bp. We annotated 102-116 total NUMTs in each diploid genome, consisting of 105,585–150,107 total bases (supplementary table S3, Supplementary Material online). The number of heterozygous NUMT insertions (those occurring on only one haplotype) varied widely, ranging from 13.2% in Gyr-1 to 55.2% in the kestrel with an average of 27.9% across all genomes; the majority of NUMTs were found on both haplotypes of all genomes except the common kestrel. The proportion of NUMTs with syntenous insertions in other genomes was relatively invariant in the large falcons with a range of 55.5–72.9% of NUMTs and an average of 61.5%. The exception to this was again the kestrel, in which only 20.7% of NUMTs had shared synteny with other genomes. We conducted a phylogenetic analysis of NUMTs by clustering them at 80% similarity, aligning clusters with mitochondrial genomes of taxa from across avian evolution, and constructing maximum-likelihood trees from each cluster. NUMTs formed 48 clusters at 80% similarity. Across all clusters, trees were generally grouped by synteny (generalized linear models: weighted-mean-of-*R*^2^ = 0.729; weighted-standard-deviation-of-*R*^2^ = 0.051). The length of NUMTs was also overwhelmingly explained by synteny (generalized linear models: weighted-mean-of-*R*^2^ = 0.81; weighted-standard-deviation-of-*R*^2^ = 0.036). Phylogenetic analysis suggests that the NUMTs found in falcons have been inserted from across the evolution of Aves, with a small number that have inserted before the divergence between the ancestors of Neoaves and Palaeognathae ∼72 Ma (fig. 5). Out of 900 NUMTs, we were able to determine the evolutionary origin of 571 using a phylogenetic analysis. The majority (*N* = 454) of these NUMTs have inserted since the divergence of Falconidae from other Neoaves in the late Paleocene (∼56 Ma) with large burst of NUMT insertions predating divergence of the ancestors of Falconinae (i.e., the “true” falcons and kestrels) and Polyborinae (i.e., the caracaras) from Herptherinae (i.e., the laughing and forest “falcons”), and another burst of insertions predating the divergence of Polyborinae from Falconinae. In total, 158 NUMTs clustered with or within *Falco* with the majority (*N* = 99) clustering most closely to a kestrel mitogenome.

We examined the propensity of NUMTs to insert into microchromosomes versus larger chromosomes by comparing the proportion of NUMTs inserted into 100 kB windows of each chromosome type with the proportion of 100 kB windows annotated as belonging to each chromosome type. We found no NUMTs that had inserted onto microchromosomes in falcons, constituting a significant propensity for NUMTs to insert onto larger chromosomes (binomial exact test: *P* = 0.002478, *N* = 158; odds ratio 95% confidence interval: 0.0–0.02307; null probability: 0.02755). We also compared the proportion of NUMTs inserted into 100 kB windows of each chromosome type before the divergence of falcons with the proportion of 100 kB windows estimated to have ancestrally belonged to each chromosome type. We again found no NUMT insertions onto ancestral microchromosomes, signifying a significant propensity for NUMTs to insert onto larger chromosomes (binomial exact test: *P* < 1e−6, *N* = 413; odds ratio 95% confidence interval: 0–0.00889; null probability: 0.1016). We find that newer NUMT insertions are significantly longer than older NUMT insertions using two independent approaches: we find that the length of NUMTs are negatively correlated with the distance of NUMTs from the mitochondrial genome of the falcon in which they were detected (generalized-linear-model-with-Poisson-error-distribution: *P* < 1e−6; parameters estimate: −0.381 bp per substation per base pair); insertions within *Falco* were also significantly longer than insertions in the vicinity of divergence from other Falconidae, other Neoaves, and Galloanserea (Bonferroni-corrected-pairwise-Wilcoxon-rank-tests: *P* < 0.0001).

### Genomic Stability and Disequilibrium

To assess whether falcon genomes were in AT–GC equilibrium, we analyzed SNVs private to each falcon genome (supplementary fig. S6A, Supplementary Material online). SNVs were called using MUMmer alignments to the kestrel genome. Focusing on private mutations effectively used a maximum-parsimony approach for assigning ancestral state, and avoided pseudoreplication of the same mutation events across multiple genomes. As parsimony approaches can be biased by branch length, we excluded the common kestrel from these analyses. This approach should strongly bias our analyses toward more recent mutations. Focusing on SNVs that are unique to each genome may also bias our analysis slightly in the direction of sequencing error. We note that Novaseq base calling errors have a slight bias (<20% higher likelihood of error) toward higher GC content (Ma et al. 2019). This may conceal some loss of GC content in our analysis, but overall low error rates (<1e−4) in SNV calls should make the impact of miscalls on our analysis negligible. The odds ratios of sites shifting from A or T to G or C were compared. We compare unique fixed and heterozygous mutations in different analyses: fixed mutations should provide a more accurate estimate of historical equilibrium; heterozygous mutations may provide an estimate of more recent mutational bias and present an upper bound on mutational bias in the absence of the full effects of gene conversion. Analyses were performed with and without CpG sites included to assess the impact of CpG hypermutability on genomic equilibrium; analyses without CpG excluded any sites that contained CpG in any of the eight genomes. Across large falcons, we found that substitutions were out of AT–GC equilibrium and biased toward increasing AT content with w/CpG sites included for both fixed (odds ratio = 1.386; χ^2^ = 2794.3; *P* < 2.2 × 10^−16^) and heterozygous (odds ratio = 1.607; χ^2^ = 50,203; *P* < 2.2 × 10^−16^) substitutions. Substitutions were also biased toward higher AT content w/o CpG included, for both fixed (odds ratio = 1.171; χ^2^ = 391.18; *P* < 2.2 × 10^−16^) and heterozygous (odds ratio = 1.082; χ^2^ = 855.43 *P* < 2.2 × 10^−16^) sites, although on a diminished scale. All individual large falcon assemblies showed a genome-wide bias toward higher AT sites with and without CpG sites included for both fixed and heterozygous sites, although an interaction was observed between fixation and inclusion of CpG sites (supplementary fig. S6B, Supplementary Material online): we observed a higher substitutional bias toward AT relative to fixed sites w/CpG included and lower bias toward AT content relative to fixed sites when CpG sites were excluded. We calculated expected equilibrium GC content with CpG sites and fixed unique substitutions for each assembly: estimated equilibrium GC content ranged from 40.12% in Gyr-2 to 42.85% in Gyr-1 with an average of 41.56% (supplementary table S4, Supplementary Material online). Estimated equilibrium points ranged from 2.13% to 0.49% below the current estimated GC content for most assemblies, with the exceptions of the lanner falcon and Gyr-1 which had a predicted equilibrium GC content of 0.72% and 0.58% higher than current levels, suggesting that biased gene conversion may drive slight increases in the GC content of these genomes despite a historical bias in fixation of higher AT content. When AT–GC equilibria were calculated using mutations with CpG sites excluded, equilibrium GC contents averaged 3.76% with a range 1.79–4.49% above current GC content, reflecting the important role of CpG hypermutability in maintaining lower GC content in falcons (supplementary table S5, Supplementary Material online). When equilibrium GC content is calculated for heterozygous sites with CpG included, equilibrium GC content is lower than current levels in all assemblies by an average of 4.01%, reflecting the importance of biased gene conversion in counteracting GC loss. When heterozygous mutations are examined with CpG sites excluded, the average estimated equilibrium GC content across large falcons is 47.8%, 5.68% above the average estimated GC content, demonstrating the important contributions of GC-biased gene conversion and CpG hypermutability in driving GC content in falcon genomes.

We assessed insertion and deletion variants to determine if genome size was in equilibrium using only insertion and deletion sites that were private to each genome, in a manner that paralleled our handling of substitutional biases in SNVs above. Among small variants, insertions were significantly longer than deletions for all peregrine falcons and *Hierofalco* genomes (Welch-*t* = 39.395; *P*  <  1e−6), but shorter than deletions in the kestrel genome (Welch-*t* = 39.395; *P*-value < 2e−16; supplementary fig. S7, Supplementary Material online). Insertions were also significantly more common than deletions for all large falcon genomes (binomial exact tests: *P* < 1e−6); the common kestrel was excluded from this analysis due to the constraints of parsimony and its deep divergence from the other falcons. Similar results were found for structural variants: insertion variants were both significantly more numerous (*P* = 0.00152) and had significantly more base pairs than deletion variants (*P* < 1e−6). Segmental duplications were significantly more numerous than segmental deletions (*P* < 1e−6), but no differences were found in their average size.

Finally, we assessed genomic equilibrium in the context of isochores and ancestral and current chromosomal states, as delineated by 100 kB windows (Costantini and Musto 2017). We assigned current chromosome states of windows based on conserved alignment to chromosome-scale assemblies for the peregrine falcon and gyrfalcon (supplementary table S6, Supplementary Material online). We assigned ancestral chromosomes states of windows based on their universally conserved alignments to chromosomes of a given type in select chromosome-scale assemblies sampled from the other lineages of Australaves (i.e., seriemas, parrots, and song birds), using the: red-legged seriema (Cariamiformes), kakapo (Psittaciformes), and Swainson’s thrush (Passeriformes). GC content (log-transformed ANOVA: *F* = 5485.723; *P* < 2e−06), CpG content (log-transformed ANOVA: *F* = 252.95; *P* < 2e−6), and the bias of unique fixed mutations toward AT with CpG sites included (log-transformed ANOVA: *F* = 22.53; *P* < 2e−6) differed significantly across windows based on current and ancestral chromosome types. Fixed unique mutations did not show a differential bias by current or ancestral chromosome types when CpG sites were excluded from the analysis (log-transformed ANOVA: *F*  =  0.1; *P* = 0.99). GC content was strongly correlated to CpG content (log-transformed ANOVA: *F* = 1831; *P* < 2e−6) and substitutional biases toward higher AT content with CpG sites included (log-transformed ANOVA: *F* = 27.48; *P* < 2e−6). Differences in CpG content were still observed between windows of differing current and ancestral chromosome states after controlling for the effect of GC content (log-transformed ANOVA: *F* = 28.37; *P* < 2e−6) by regressing it out. However, differences in fixed substitutional bias (w/CpG) toward higher AT were not observed after controlling for GC content (log-transformed ANOVA: *F* = 5.828; *P* = 0.00295), indicating that these trends were driven primarily by GC content rather than chromosomal traits.

**Fig. 6.**
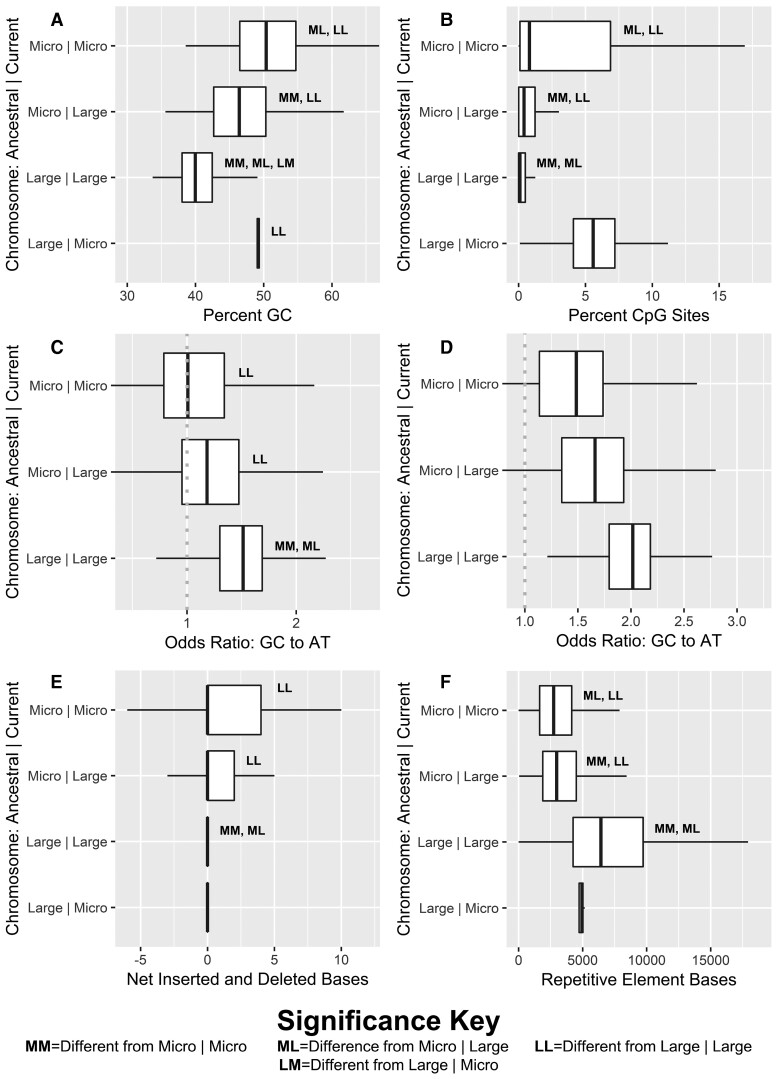
Boxplots of genomic features by Ancestral|Current chromosomal state of 100 kB windows across the eight falcon genomes for: (*A*) percent GC content; (*B*) percent CpG sites; (*C*) odds ratios of AT to GC mutation with CpG sites included; (*D*) odds ratios of AT to GC mutation with CpG sites excluded; (*E*) net inserted and deleted bases; (*F*) total number of bases annotated by as belonging to TEs. Significant differences are denoted with letters according to descriptions in the bottom key.

Regions conserved on microchromosomes (Micro|Micro) were found to have higher GC content (*P* < 2e−6), CpG content (*P* < 2e−6), and substitutional bias toward higher AT content when CpG sites were included (*P* < 2e−6), relative to regions conserved on large chromosomes (fig. 6; supplementary figs. S8–S11, Supplementary Material online). They also displayed significantly higher levels of GC content (*P* < 2e−6) and CpG content (*P* < 2e−6) than regions ancestrally located on microchromosomes and fused to large chromosomes, indicating that fusions of microchromosomes to larger chromosomes have resulted in reduced GC and CpG content over time. Regions ancestrally located on microchromosomes and fused to larger chromosomes showed a bias of fixed mutations toward higher AT content relative to regions conserved on large chromosomes (*P* < 2e−6), but where not differentially biased in unique fixed mutations relative to regions conserved on microchromosomes.

To determine if observed biases in inserted base pairs were related to chromosomal fusions, we assess net inserted or deleted base pairs across 100 kB windows in the context of current and ancestral chromosomal state (log-transformed ANOVA: *F* = 9.768; *P* < 2e−6; fig. 6*E*; supplementary fig. S12, Supplementary Material online). Regions formerly on microchromosomes and fused to large chromosomes had more inserted bases than windows conserved on large chromosomes (*P*  =  0.000064), as did windows with conserved positions on microchromosomes (*P* = 0.00125). But, no significant difference was observed between windows formerly on microchromosomes that had fused with larger chromosomes and those that had not. Model selection criteria did not indicate GC content as important to this model so it was not included. The total number of small indels was, however, strongly correlated to GC content (*P* < 1e−6). After controlling for GC content, we found that windows ancestrally belonging to microchromosomes had significantly more indels than those that belonged to large chromosomes (*P* < 1e−6). While there was a trend toward more indels on windows that had remained on microchromosomes relative to windows that had fused to large chromosomes from microchromosomes, this trend was nonsignificant after controlling for multiple tests (*P* = 0.002). Counts of unique large structural variants were generally independent of both current and ancestral chromosome state, but were significantly depleted in regions formerly on microchromosomes and fused to large chromosomes relative to regions conserved on large chromosomes after controlling for GC content (*P* = 0.00009).

Finally, we looked at changes in chromosome state and base pairs annotated as belonging to transposable elements (TEs) across windows (log-transformed ANOVA: *F* = 1566; *P* < 1e−6; fig. 6*F*; supplementary fig. S13). Regions conserved on microchromosomes had fewer repetitive bases than those conserved on larger chromosomes and those formerly belonging to microchromosomes but fused to large chromosomes had intermediate numbers of repetitive bases (*P* < 1e−6) to either. Higher GC content was found to be strongly negatively correlated with TE base content (*P* < 1e−6), and the effect of GC content was regressed out (log-transformed ANOVA: *F* = 300.5; *P* < 2e−6; fig. 6*F*; supplementary fig. S14). After controlling for GC content, we found that windows ancestrally belonging to microchromosomes have fewer repetitive bases than those conserved on larger chromosomes (*P*-value < 1e−6). However, windows formerly belonging to microchromosomes but fused onto large chromosomes were nonetheless depleted in repetitive bases relative to those conserved on microchromosomes (*P*-value  =  0.0005). In light of a confirmed loss of GC content, this reversal of relationships when accounting for GC content suggests a more rapid loss of bases associated with TEs than GC content on regions of microchromosomes that have fused to large chromosomes.

## Discussion

### Overview

We sequence the genomes of eight falcons using 10X Genomics linked-reads and produce highly-contiguous scaffold-level assemblies that exceed de novo assembly contiguity for all previously sequenced falcon species other than the gyrfalcon and the common kestrel, and at assembly sizes comparable to those reported for all previously sequenced falcons. We find genetic variation, particularly SNVs, to be highly variable across species with a ∼7X greater distance between heterozygous sites in gyrfalcons relative to the common kestrel. Using the Trinnotate pipeline (Bryant et al. 2017), we annotate a number of genes comparable to those found in previously published falcon genomes. We find distributions of GC content and isochore families similar to those reported in other birds. Bird genomes resembled mammalian genomes most closely, but were shifted toward higher GC content as reported in previous studies (Constantini and Musto 2017). We annotate ∼6.5% of the falcon genomes as belonging to repetitive elements, a number comparable to reference-based analyses of saker and peregrine falcon genomes (Zhan et al. 2013) and higher than a previous de novo annotation of the peregrine genome (Zhang et al. 2014). We also find broad evidence for a lack of repeat activity in falcons. This finding is consistent with previous reports—using standardized methodology—that show falcons to be in the lowest quartile for repeat content in birds (Zhang et al. 2014). These findings are in keeping with previous work showing highly variable levels of TE activity across birds, but at odds with reports of enriched TE activity in bird groupings that have undergone recent speciation (Galbraith et al. 2021). We also find simple repeats corresponding to interstitial telomeres. While interstitial telomeres are known to be common in ratites (Palaeognathae), land fowl (Galloanserae), and waterfowl (Order: Anseriformes), they are not known to be common in the clade consisting of falcons, parrots, seriema, and songbirds (Nanda et al. 2002).

### Phylogeny and Demography

We construct a maximum-likelihood phylogeny based on all variable sites and timescale the phylogeny based on the divergence of the common kestrel from larger falcons. We find divergence times that are generally more recent than previously reported among large falcons with a split between peregrine falcons and *Hierofalco* of 588 kya. This contrasts with an estimated divergence time of ∼2 Ma based on an eight loci and partial mitochondrial tree (Fuchs et al. 2015) and an ∼4 Ma based on genome-wide 4-fold degenerate sites among diapsids (Zhan et al. 2013). Our tree differs from other trees in both the type of data used and in its calibration point. We calibrate using the split of old-world kestrels from other falcons, a more recent calibration point than the splits between Falconinae and Polyborinae (∼16.3 Ma) and the laughing falcon and forest falcons (∼20 Ma) used by Fuchs et al.. Our calibration point was much more recent than the split between Lepidosaurs and Archosaurs (∼300 Ma) and Neoaves and Galloanserae (∼70 Ma) used by Zhan et al. We note a trend across all of these previous studies that more recent calibration points produce more recent divergence times, and it is possible that our more recent calibration point contributed to our findings of more recent divergence.

We conduct PSMC analyses to assess changes in the population sizes of falcons over time. Our findings suggest that the population sizes of large falcons and the common kestrel have generally been low but increased substantially within the last few thousand years before undergoing more recent population declines. Gyrfalcons are an exception to this trend and have been undergoing steady population decline since their divergence from the saker falcon. These population increases generally coincide with both the recent deglaciation event at the end of the Pleistocene and the onset of human agriculture with the beginning of the Neolithic era in human development. The exact reasons for the population increases cannot be determined, but in light of the long history of human–falcon interactions (Wilcox et al. 2019) and the known importance of grasslands to falcon ecology and evolution (Fuchs et al. 2015), both humans and climate change have likely contributed to this increase. Falcons are likewise known to have undergone more recent population declines associated with a variety of human activities ranging from collection for falconry, hunting, and organochloride poisoning. The lanner falcon and Eurasian peregrine falcon show population increases and subsequent bottlenecks on the order of 160 and 50 kya, respectively. The older lanner falcon expansion generally coincides with a previous deglaciation event (Wunsch 2004). We have no explanation for the apparent expansion of the Eurasian peregrine. Ultimately, studies on more falcon species and more genomes may provide additional insights into the underlying reasons for falcon population fluctuations in the more recent and distant past (Spence et al. 2018; Gu et al. 2021).

### Nuclear mitochondrial DNA segments

We also annotate several dozen NUMTs in each falcon genome. While we annotate somewhat more NUMTs, these findings are consistent with previous reports from falcons (Nacer and do Amaral 2017), and are higher than typical of other birds (Liang et al. 2018). Most but not all NUMTs shared synteny with NUMTs in other genomes. Phylogenetic analysis also showed that all NUMTs grouped most closely with syntenous NUMTs when these occurred. These findings suggest that primary insertion is the overwhelming source of NUMTs in falcons, with secondary duplication playing only a minor role. We find strong evidence that the NUMTs found in falcons have been accumulating across avian evolution, with the earliest likely predating the split between Ratites and other birds. However, most NUMTs inserted within the early evolution of Falconidae. This could suggest something particular to Falconidae that favored NUMT insertion, but could also suggest slow but continual decay of older NUMTs within the genomes of falcons and their ancestors. Genomes from other Falconidae will undoubtedly help to clarify these possibilities when they become available. Insertions within falcons are also ongoing. We find support for the hypothesis of an ongoing reduction in length of NUMT insertion over time, possibly favored by selection for smaller genomes in birds (Kapusta et al. 2017). We also propose—based on our findings of large insertions in large falcons but not the common kestrel—that NUMT insertion may be more common and larger in species with smaller effective population sizes, as smaller population sizes should increase the likelihood of genomic fixation for novel insertions due to drift and decrease the efficiency of selection against such insertions. This could explain higher numbers of NUMTs in the carnivorous Falconidae lineage, but this is a hypothesis better analyzed across a large number of species. NUMTs were biased against insertion on microchromosomes, and as such, microchromosome loss could favor NUMT insertion. However, given the large accumulation of NUMTs that appear to predate the divergence of falcons from caracaras (and therefore the loss of microchromosomes in falcons), higher numbers of NUMTs within falcons cannot be generally attributed to their unique chromosomal rearrangements.

### Chromosomal Fusions and a Genome in Flux

We find that falcon genomes are out of AT–GC equilibrium and that they are losing GC content. The loss of GC content appears to be genome-wide, but, as reported in several mammals (Duret et al. 2002), it is most pronounced in high-GC regions. Moreover, we find evidence that the loss of GC content is linked to the loss of microchromosomes in falcons: regions of microchromosomes that have fused to larger chromosomes are depleted in GC content relative to regions that have remained on microchromosomes; and regions of microchromosomes that have fused to larger chromosomes are losing GC content at a higher rate than typical of the larger chromosomes on which they now occur, even when controlling for higher GC content. Conversely, regions of larger chromosomes that have merged to microchromosomes appear to have gained GC content, although these sites were too rare to assess recent substitutional biases. The historic loss of GC content is paralleled by a loss of CpG islands, although the ongoing loss of GC content is driven largely but not exclusively by the loss of CpG sites. Fixed differences should generally be older than heterozygous differences. By comparing odds ratios across unique heterozygous and homozygous SNVs, we gain further insights into differences in substitutional biases over time. These findings support historical and ongoing GC loss. The observation of highest GC loss among heterozygous sites with CpG included highlights a particularly important role for CpG hypermutability in driving current GC loss in falcons. The relatively low but significant loss of GC at heterozygous sites with CpG excluded, particularly relative to fixed sites with CpG excluded, suggests that the pace of GC loss at non-CpG sites has likely slowed over the history *Falco* evolution.

Overall, our findings provide support for the BGCH (Duret et al. 2006) as a driver of genomic substitutional disequilibrium in falcons. Microchromosomes that have fused to larger chromosomes are presumably subject to reduced recombination rates as a result of a greater number of base pairs over which recombination events may occur (Ellegren 2010; Perry et al. 2020; Schield et al. 2020). Reduced recombination events should result in a loss of GC-biased gene conversion on former microchromosomes (Duret et al. 2006; Ellegren 2010), and with it a lower GC content on these regions. Conversely, the smaller regions that have translocated from larger chromosomes onto microchromosomes are gaining GC content as a result of increased recombination and the GC-biased conversion that comes with it. As fused regions appear to be intermediate to conversed regions in GC content, it is plausible that these regions are still moving toward a new equilibrium. The more perplexing issue is the seemingly genome-wide loss of GC content. Fusions of chromosomes are themselves a genome-wide phenomenon: while not all fusions have resulted in a change of chromosome size categories, we would expect all fusions to have increased chromosome size. As such, genome-wide AT–GC disequilibrium may be driven in part by an overall reduction in recombination rate. More detailed studies on specific chromosomal rearrangements within Falconidae, and potentially other birds (Kretschmer et al. 2021), will be necessary to explore this hypothesis.

We likewise detect a bias toward inserted bases relative to deleted bases in both small indels and large structural variants. These findings are in keeping with previous reports that falcons have the lowest microdeletion rate observed in birds (Kapusta et al. 2017), and may also explain the longer genes, intragenic spaces, and introns in falcons than typical of other birds (Zhang et al. 2014). As biased gene conversion has been shown to favor inserted bases over deletions, this result is at odds with our findings of AT-biased substitutional and suggests that other mechanisms may be important toward driving insertions (Leushkin and Bazykin 2013); however, the accumulation of insertions on former microchromosomes irrespective of current state likely reflect higher ancestral recombination rates. While insertion-biased gene conversion has been previously reported to account for interspecific fixations, our findings demonstrate that it can be observed intraspecifically as well. Importantly, indels and large structural variants seem to comprise a genomic feature for which ancestral chromosome types seem to be more important than current chromosome type. As such, conserved sequence motifs or other factors that intrinsically separate microchromosomes and macrochromosomes may remain on these fused regions and continue to affect structural variation (Perry et al. 2020).

Analysis of TEs and chromosomal type confirms that repetitive elements generally tend to accumulate on larger chromosomes, as previously proposed (International Chicken Genome Sequencing Consortium 2004; Boissinot et al. 2019). Interestingly, regions of microchromosomes that have fused to larger chromosomes show intermediate levels of bases annotated as originated from repetitive elements. Given multilateral evidence for a dearth of repeat activity in falcons, it is probable that the intermediate levels of bases originating among repetitive elements are the result of reduced efficiency at removal of older repetitive bases on larger chromosomes. This finding is again consistent with lower recombination rate, as ectopic recombination typically drives removal of repetitive elements (Frahry et al. 2015; Ji and DeWoody 2016; Jedlicka et al. 2020). A lack of repeat activity may itself be considered an additional form of genomic instability. The extent to which falcons have experienced inactivation of repeats requires further study. Repeat activation is often associated with speciation events (Jurka et al. 2011), but the falcons that we sampled have undergone rapid radiations without any evidence for enhanced repeat mobilization. It is tempting to speculate on a special role for the reduction of repeat activity in falcon evolution, given the links between repeat content, radiations, recombination, and chromosome stability and the peculiar nature of falcons in all these areas.

### Conclusions

Taken together, our findings provide evidence that falcon genomes have undergone a dramatic reduction in recombination rate that has thrown them out of AT–GC equilibrium and favored the accumulation of AT content. We link these findings in part to the substantial number of chromosomal fusions and microchromosome loss in falcons. While differences between microchromosomes and macrochromosomes have been well characterized in the past (Ellegren 2010; Perry et al. 2020), little is known as to whether these differences are the result of chromosome size or sequence motifs. Our findings suggest that differences in base composition, recombination, and TE content between microchromosomes and larger chromosomes are largely products of chromosome size, and subject to disequilibrium following size changes. Conversely, structural variant and indel formation may be driven in part by factors other than size. It has been previously asserted that differences in genomic stability account for the differences in substitutional bias between mammals and birds (Mugal et al. 2013) and our results support this assertion. Ultimately, our results show that falcon genomes are undergoing similar genomic changes to many mammals following similar (albeit) lesser microchromosome loss events and support a strong role for biased gene conversion in both maintaining and driving genomic equilibrium and disequilibrium.

## Materials and Methods

### DNA Extraction and Sequencing

Blood samples were obtained from female falcons of known ancestry during the course of normal veterinary care (table 1). Samples were collected in accordance with IACUC protocol FS 18-0001. High-molecular weight DNA was extracted from samples using the Qiagen MagAttract Extraction kit (Hilden, Germany) as per the manufacturer’s instructions, with an additional tissue lysis step to improve yield. DNA fragment size was verified to be ∼50 kb using pulse-field gel electrophoresis on a Bio-Rad Laboratories CHEF Mapper XA System (Hercules, CA, USA). Libraries were prepared using the 10X Chromium Genome High Throughput (HT) Gel and Bead Kit Version 2 (Pleasanton, CA, USA), barcoded with the Chromium i7 Multiplex kit, and sequenced on an Illumina Novaseq6000 using the NovaSeq6000 S4 300 Cycle Reagent Kit (San Diego, California, USA). Sequence quality was assessed using FastQC version v0.11.8 (Andrews 2017), and sequences were assessed for potential GC bias and contamination with Kat version 2.3.1 (Mapleson et al. 2017).

### Genomic Assembly

Sequences were assembled using the Supernova assembly software for de novo assembly of linked-reads (Weisenfeld et al. 2017). Default settings were used, and sequences were not trimmed beforehand in accordance with the instructions of the software provider. In the case of the Black Shaheen, raw reads were subsampled down to ∼77X coverage based on the manufacturer’s instructions. Supernova provides several FASTA output options. Genomes were output using the “pseudohap2” option to produce two-phased haplotypes for each genome. Downstream analyses either used both sets of phased haplotypes to perform analyses on each diploid genome or used single representative scaffolds from each phased haplotype to analyze haploid genomes. Scaffolds smaller than 16 kb were discarded from all assemblies.

Assembly completeness was assessed using the BUSCO version 5.0 and the “aves_odb10” database and Augustus 3.4 to search for conserved avian single-copy orthologs (Simão et al. 2015). BUSCO was run using an initial chicken model for protein structure and supplemented with a new search using a genome-specific AUGUSTUS model based on discovered genes (option “long”).

The presence of Z and W sex chromosomes were identified by searching for diverged CHD-Z and CHD-W Chromosome Helicase genes (Fridolfsson and Ellegren 1999) with HMMER (Wheeler and Eddy 2013). In brief, HMMER profiles for both genes were created by downloading all versions of these genes from birds between 200 and 2,200 bp in length from NCBI Genbank (Benson et al. 2012) using “esearch” from the Entrez tool utilities (Kans 2017) on February 7, 2020, with the following specific search parameters:

txid8782[Organism:exp] AND biomol_genomic[PROP] AND (“200”[SLEN]: “2200”[SLEN]) AND chd-z[All Fields] OR chd-w[All Fields]

These were aligned using a MAFFT version 7.407 (Katoh and Standley 2013) iterative global alignment and used to construct an HMMER profile which was used with nHMMER to search for copies of these genes at an *e*-value threshold of 0.000001. Results were manually confirmed as CHD-Z and CHD-W with NCBI BLAST searches.

Mitogenomes were created by aligning raw reads to consensus genomes of archival mitogenomes. In brief, all mitogenomes for peregrine falcons, *Hierofalco*, and the common kestrel were downloaded from NCBI GenBank using E-utilities with the following respective search parameters:

txid8954[Organism] mitochondrion[filter] AND (“16000”[SLEN] : “20000”[SLEN])
(txid345155[Organism] OR txid120794[Organism] OR txid345164[Organism]) mitochondrion[filter] AND (“16000”[SLEN] : “20000”[SLEN])
txid100819[Organism] mitochondrion[filter] AND (“16000”[SLEN] : “20000”[SLEN])

Archival mitogenomes were aligned using a Mafft local-pairs iterative alignment and constructed into an HMMER database. Archival mitogenomes were then aligned back to these preliminary consensus genomes with HMMER to determine common starting points. Mitogenomes were reoriented to the same starting point, realigned with a Mafft local-pairs iterative alignment and final archival consensus genomes were created. Long Ranger was used to align raw reads back to the appropriate archival consensus mitogenome (i.e., Peregrine, *Hierofalco*, Common Kestrel) for each sequenced genome with the requirement that reference alleles have a minimum depth 3X higher than the reference allele.

### Variant Calling

Variant calling was performed using several methods. First, 10X Genomics Long Ranger (Marks et al. 2019) was used to align raw demultiplexed reads from each sample back to their own haploid assemblies. In accordance with the proprietor’s instructions, GATK (version 3.5) was used as a variant caller with all 10X quality filters enabled. These filters excluded variants with: total Pfred quality <30; Pfred Quality of < 15 for any heterozygous allele; an allele fraction <15%; inconsistent phasing (based on linked reads); >3 bp unphased homopolymer insertions (based on linked reads); and poorly aligned variants calls of the whole molecule relative to the scaffold (“10X_RESCUED_MOLECULE_HIGH_DIVERSITY”). While this approach worked well for calling variants within each genome, it was insufficient for alignments across falcon species due to higher sequence divergence. As such, MUMmer version 3.2.6 (Kurtz et al. 2004) was used to align whole diploid genomes from each assembly to the haploid common kestrel genome. To facilitate alignments between species, MUMmer alignments used the NUCmer algorithm with a minimum alignment length of 100 bp and a maximum gap of 500 bp within a single alignment, in accordance with manual guidelines for “fairly similar sequences.” The common kestrel genome was chosen as a common genome for alignment due to its equal evolutionary distance from all other genomes, thereby avoiding biases in variants calls resulting from better alignments between more closely related species. Custom scripts were used to convert MUMmer alignments of small variants into vcfs and to annotate larger variants into bed files. To validate this approach, phased haplotypes of each diploid genome were aligned back to one another using MUMmer and the results were compared with the results of the Longranger output. Target haploid genomes for all alignment approaches had scaffolds smaller than 100 kB removed to better facilitate large structural variant analysis. Large structural variants were annotated from MUMmer alignments to the common kestrel and between alternative pseudohaplotypes of the same genomes using a custom script. Large insertions and deletions were defined as a 50 bp or greater breaks in alignment on one sequence, without any break in alignment on the other, with large deletions demonstrating the break on the reference scaffold and large insertions demonstrating the break on the aligned scaffold. Large inversions were defined as 50 bp or greater reverses in direction of alignment between scaffolds, relative to the majority rule directionality of alignments up to that point between the same scaffolds. Large insertions, deletions, and inversions were all filtered to exclude any matches to duplicated regions. However, segmental deletions and duplications were defined as 50 bp or greater matches in alignments to single proximally aligned positions: segmental deletions were annotated when a tandem alignment of the reference genome matched a single upstream position of the aligned genome; segmental duplications were annotated where tandem regions of the aligned genome matched a single proximal region of the reference genome.

Small insertions and deletions are insertions and deletions of <50 bp relative to the reference genome after normalization. Small insertions and deletions were left aligned and normalized prior to any analysis. All comparisons and processing of variants were performed using Samtools and Bedtools (Li et al. 2009; Quinlan and Hall 2010; Li 2011).

### Transcriptome and Gene Annotation

Samples were collected in RNA*later*® (Thermo Fisher Scientific: Waltham, MA, USA) from a female gyrfalcon immediately following clinical euthanasia for amyloidosis of the liver and frozen at −80 °C until processing. In total, samples were taken from 22 distinct tissues: the liver, pancreas, chest muscle, stomach, hyperpallium (brain), cerebellum (brain), thyroid, gizzard, heart, kidney, lung, spleen, and at 10 cm intervals in the intestine (with 10 intestinal samples total). All samples were extracted using the Qiagen RNA extraction kit, along with two negative controls—one taken from the beginning and one at the end of all extraction steps. Extracted RNA was normalized to 10 ng in 50 µl for library preparation. Library preparation was performed with the NEB Ultra II RNA Library Prep kit and sequenced in multiplex on a NovaSeq 2 × 150 flowcell.

Reads were used to produce a de novo transcriptome. In brief, reads were mapped to the Canadian gyrfalcon reference genome (Gyr-1) using HiSat2 (Kim et al. 2019) and assembled into transcripts using StringTie (Pertea et al. 2015). Transcript regions were identified with Transdecoder (Haas et al. 2013), and the transcriptome was annotated using the Trinotate pipeline (Bryant et al. 2017). This transcriptome was then mapped to each genome to produce gene annotations using the “merge” option in stringtie. Gene ontology (GO Term) annotations were produced using BLAST2GO (Conesa et al. 2005).

### Repeat Annotation

Repeats were annotated using RepeatModeler2 (Flynn et al. 2020) and RepeatMasker (Tarailo-Graovac and Chen 2009). RepeatModeler2 was run independently on each diploid falcon genome using rmBLAST as a search engine and the LTR structural modeling enabled. The default max sample size of 243,000,000 was used for a total number of 403,000,000 bases sampled across all rounds; this had the effect of sampling ∼16.8% of each haploid falcon assembly. The same methods were used to produce repeat libraries for other Inopinaves genomes for comparison, with the modification that genome max sample size was set to only 123,000,000. Falcon consensus repeat libraries were further processed by merging and clustering outputs across all assemblies. In total, 2,643 repeat families were detected across all eight diploid assemblies. These were filtered using Usearch “sortbysize” to remove all repeat families with <20 copies found in any given genome, leaving 1503 putative repeat families. These were pooled, sorted by length and clustered into merged consensus (“consout”) sequences at 80% (subfamily level) with USEARCH (Edgar 2010) using the “cluster_fast,” resulting in 955 unique clusters. These were further filtered to remove any subfamilies that had not been reported in at least 40 copies across all genomes, leaving 238 consensus subfamilies. These were annotated according to the initial seed sequence used to produce the cluster with a custom bash script. Repeatmasker v4.1.2 was run on each diploid falcon genome using rmblast v2.11.0 as a search engine and the merged falcon consensus subfamily sequences as a custom library. The “-gccalc” option was enabled to correct for background GC levels in target sequences and the “-s” option for a more sensitive search. Alignments were saved and used to create a divergence curve using the included RepeatMasker utilities. To date past TE activity, the same repeat library and annotation methods were used to annotate additional avian genomes: the emu (GCA_016128335.1); the red jungle fowl (GCF_000002315.6); the red-legged seriema (GCA_009819825.1); the Swainson’s thrush (GCF_009819885.1); and the kakapo (GCF_004027225.2).

We searched for active repeats comparing polymorphic structural variants against repeat annotations using Bedtools: a repeat was considered polymorphic if it made up at least half of a polymorphic insertion structural variant and was contained entirely within this insertion structural variant. We searched for active repeats by searching for intact ORFs. In brief, NCBI E-Utilities “esearch” was used to download intact repeats belonging to the LINE, LTR, and DNA repeat subfamilies according to the following search criteria:

LINES:
“reverse transcriptase” AND complete[Title] AND (non-LTR[Title] OR LINE[Title] OR “Chicken Repeat 1”) AND (“1200”[SLEN] : “8000” [SLEN]) NOT “pseudogene”
LTRs:
“reverse transcriptase” AND “complete” [Title] AND “LTR” NOT non-LTR AND (“1200” [SLEN] : “12000” [SLEN]) NOT “pseudogene”
DNA:
“complete” [Title] AND Transposase AND (Harbinger OR hAT OR MULE) AND (“1200” [SLEN]: “10000” [SLEN]) NOT “pseudogene.”

In total, 66 LINE sequences, 122 LTR, and 106 DNA sequences were acquired. Each of these libraries was aligned using Mafft l-insi iterative local-pairs alignments with up to 1,000 iterations, and constructed into HMMER databases, which were used to extract repeat ORFs with nhmmer. We considered repeat ORFs to be potentially active if they exceeded minimum length requirements for active ORFs of their subclass based on reports from NCBI GenBank: 2,000 bp for LINEs; 2200 bp for LTRs; and 1,200 bp for DNA transposons.

### Isochore Window Annotation

Genomes were divided into nonoverlapping 100 kB windows and nonoverlapping 1 MB windows. Base composition, CpG sites, variants, and repeats were assessed for each window (Costantini and Musto 2017). Windows were assigned as belonging to ancestral chromosomal states based on their alignments to chromosome-scale NCBI Genbank/RefSeq genomes for the Swainson’s thrush (*Catharus ustulatus*: GCF_009819885.1), kakapo (*Strigops habroptila*: GCF_004027225.2), and red-legged seriema (*Cariama cristata*: GCA_009819825.1). These birds were chosen to sample across all major clades related to falcons: the parrot and song bird clades of Eufalconimorphae (Suh et al. 2011), and the outgroup to this clade (Prum et al. 2015), respectively. They were also intended to sample birds across a wide range of chromosomal configuration: Swainson’s thrush has a typical avian chromosome count with 2*N* = 80 annotated autosomes. Kakapos have a reduced chromosome count with 2*N* = 46 annotated autosomes; and, the red-legged seriema has a more fractured genome with 2*N* = 100 annotated autosomes. Whole genomes from each falcon were aligned to each of these genomes using MUMmer with the NUCmer algorithm, a minimum alignment length of 100 bp and a maximum gap of 500 bp within a single alignment. Windows were assigned as ancestrally belonging to: microchromosomes if they aligned exclusively to chromosomes of <20 MB across all three nonfalcon genomes, intermediate chromosomes if aligned exclusively to chromosomes >20 MB and <40 MB across all three nonfalcon genomes, and macrochromosomes if they aligned exclusively to chromosomes >40 MB across all three falcon genomes. “Large chromosomes” were defined as any chromosome >20 MB. Window’s aligning to chromosomes of different size categories within or across the three nonfalcon genomes were classified as belonging to an ambiguous ancestral state and removed from analyses relating to ancestral state. The current state of windows as belonging to microchromosomes, intermediate chromosomes, and macrochromosomes was annotated in an identical manner, except with alignment to chromosome-scale assemblies of the gyrfalcon (GCF_015220075.1) and peregrine falcon (GCA_001887755.1) found on NCBI RefSeq and Genbank, respectively. Windows that matched different size classes in either the *Falco* or ancestral genome comparisons were excluded from further analysis of effects of chromosomal state. Windows belonging to sex chromosomes were also annotated using the same approach; as the peregrine assembly was obtained from a male, it contained only a Z Chromosome, and windows belonging to the W Chromosome could not be annotated in this way and were excluded from the windows-based analysis. All statistical analyses relating to base composition were performed on 100 kB windows in accordance with typical isochore size and standard practices for isochores annotation (Costantini and Musto 2017). Base composition and CpG sites were determined using the program SeqTK (Shen et al. 2016).

Graphs were made for falcon and other amniotes using the following archival sequences: the kakapo (GCF_004027225.2); the Swainson’s thrush (GCF_009819885.1); red-legged seriema (GCA_009819825.1); the saltwater crocodile (*Crocodylus porosus*: GCF_001723895.1); the green sea turtle (*Chelonia mydas*: GCF_015237465.1); sand lizard (*Lacerta agilis*: GCF_009819535.1); and a human (*Homo sapiens sapiens*: GCF_000001405.39).

### NUMT Annotation

NUMTs were annotated using HMMER. In brief, all mitochondrial genomes from Falconidae on NCBI were downloaded on October 4, 2021, using NCBI Entrez tools “esearch” with the search terms:

txid8949[Organism] mitochondrion[filter] AND (“16000” [SLEN] : “20000” [SLEN]).

Several additional mitogenomes were downloaded in the same manner to assure taxon-sampling across Aves: *Catharus ustulatus* (CM020378); *Cacatua alba* (MT920475); *Picus canis* (NC_045372.1); *Strix occidentalis* (MF431746); *Accipiter gentilis* (AP010797); *Gavia stellata* (AY293618); *Balearica regulorum* (FJ769841); *Columba livia* (KP319029); *Gallus gallus gallus* (AP003322); and *Struthio camelanus* (AP003322).

Mitogenomes were aligned using the Mafft local-pairs iterative alignment with up to 1,000 iterations, and aligned into an HMMER database. Falconidae mitogenomes were realigned to the HMMER database with nhmmer and then reoriented to the same start position. Reoriented mitogenome sequences were then concatenated with themselves to ensure that all regions were fully present without a break point. These concatenated and reoriented mitogenomes were then realigned with a Mafft local-pairs iterative alignment with up to 1,000 iterations, and compiled into an HMMER database. This database was used to search the full diploid genomes of all falcons with nhmmer using an *e*-value of 10^−6^. Synteny analysis was performed by converting the locations of NUMTs in all falcons to locations in the kestrel genome based on MUMmer alignments using a custom script and overlapping locations were then identified with Bedtools; NUMTs with any overlap were categorized as syntenous. Extracted NUMTs were clustered at 80% using USEARCH: in brief, NUMTs were sorted by length using USEARCH “sortbylength” and then clustered at 80% using USEARCH “cluster_fast” with “maxaccepts” set to “1” (limiting each NUMT to assignment in a single cluster) and “maxrejects” set to “0” (ensuring that all NUMTs were tested against all clusters). NUMTs were then aligned within their assigned clusters using a Mafft local-pairs iterative alignment with up to 1,000 iterations, and trees were constructed for each cluster using RAxML (Stamatakis 2014) with a GTR model, including gamma-distributed rate variation. Trees were built using the best of 5 guide trees, and bootstrapped 20 times.

### Phylogenetic Tree Construction

SNV calls from MUMmer alignments to the haploid kestrel genome were used to create aligned full-genome consensus sequences for each genome using Bcftools “Consensus” in Samtools. Heterozygous sites were coded using IUPAC ambiguity codes. These were converted to gvcfs using a custom script and filtered against conserved sites to extract aligned vcfs of variable sites in each genome. These vcfs were converted into Fasta alignments with a custom script and Fasta alignments were converted to Phylip alignments with the program Readseq (Gilbert 2003).

A maximum-likelihood tree was constructed in RAxML with all variable sites using a GTR model with gamma-distributed rate variation between sites. The common kestrel was specified as an outgroup. Bootstraps were performed in 1,000 replicates and drawn onto the best tree to assess branch support. The tree was scaled to time and dated using the program R8S (Sanderson 2003). The split between the common kestrel and other falcons was used as a calibration point. This split was specified as 7.246 Ma, corresponding to the Tortonian–Messinian junction in the upper Miocence based on the fossil record of the earliest diverged member of the old-world kestrels (Boev 2011) and in accordance with previous molecular-clock-based estimates of this split (Fuchs et al. 2015). Divergence times were estimated using the penalized-likelihood approach (Sanderson 2002) and the truncated Newton algorithm.

### Pairwise Sequential Markovian Coalescent

Analysis of falcon demography was performed using a pairwise sequential Markovian coalescent (PSMC) analysis with the program PSMC (Li and Durbin 2011). This analysis used unphased SNVs called with Longranger. The model was parametrized with a maximum coalescent time of 12 *N*_e_ generations, theta/rho of 2, and modeled across 100 time-intervals with separate consensus estimates for the first and last eight intervals and 28 three-interval segments between these. Theta/rho was estimated based on the per-generation mutation rate, mu, of 4.6 × 10e−9 estimated from the collared flycatcher (Smeds et al. 2016), and the per-generation recombination rate of 0.0014 in the zebra finch (Singhal et al. 2015). All PSMCs were bootstrapped 100 times.

Generation times were estimated using the equation: [1] *g* = *a* + [*s*⁄(1 − *s*)], where “*a*” is the age of sexual maturity and “*s*” is the expected adult survival rate (Zhan et al. 2013). This provides a generation time of 3.13 years for the common kestrel (Dijkstra et al. 1990), 6 years for the peregrine falcons (Court et al. 1989; Kauffman et al. 2003), 6.56 years for the saker falcon (Wink et al. 1999; Kenward et al. 2007), and 12 years for the gyrfalcons (Booms 2010). Reliable estimates of survival could not be found for lanner falcons, and a generation time of 6 years was used.

### Scripts and Statistical Analyses

All scripts and codes are included as Supplementary Material online. All statistical analyses were run in R Studio 1.1,1456 (R Studio, 2016) using R version 3.5.1 “Feather Spray” (R Core Team 2018). Equilibrium GC content was calculated using the following formula:

$${\mathrm{Equilibrium}}_{\mathrm{GC}}=1-OR_{\mathrm{GC}\to AT}/(1+OR_{\mathrm{GC}\to AT})$$

Isochore analysis was conducted using linear models. Models comparing current and ancestral chromosomal states were chosen using stepwise selection based on AIC values with both forward and backward selection enabled. Where genome-specific differences were detected, genomic differences were controlled for by inclusion in models as a fixed blocking effect. Where GC content was included in the model, it was regressed against the response variable to remove its effect and subsequent analyses were performed on residuals. As count data such as those used in this analysis typically has variance in proportion to mean, log_2_(*x* + 1) transformations were performed on all data sets to normalize residuals. Anderson–Darling tests were used to assess the normality of residuals and confirm the efficacy of transformations, which resulted in a ∼10–50X decrease in the size of scale of the Anderson–Darling statistic (Anderson and Darling 1954). Anderson–Darling tests were performed using the “nortest” package. Comparison of levels of between chromosomal states was performed using Tukey’s “Honest Significant Difference Method.” The odds ratios of substitutional bias across windows could be biased by values of “0” and infinity if GC to AT or AT to GC substitutions were, respectively, lacking: we addressed this by using an upper-bound substitutions rate of each type by adding a single substitution of each type to the calculation of these odds ratios. We use a false-discovery-rate-corrected alpha value of 0.0026 in accordance with a Dunn–Šidák correction of an initial alpha of 0.05 for our 20 independent statistical tests.

## Supplementary Material

Supplementary data are available from *Genome Biology and Evolution* online.

## Supplementary Material

evac090_Supplementary_Data

## Data Availability

The genome sequences have been deposited at GenBank under BioProject accession PRJNA 794321.
