# Supplementary material for: Linked-Read Sequencing of Eight Falcons Reveals a Unique Genomic Architecture in Flux
Source: Genome Biol Evol. 2022 Jun 14;14(6):evac090. doi: 10.1093/gbe/evac090 (PMC9214253; doi:10.1093/gbe/evac090)
Supplement: evac090_Supplementary_Data [file evac090_supplementary_data.zip › Supplementary figures.pdf]

Supplementary Figure 1: Heat maps of GC count (y-axis) by 27-mer frequency (x-axis) in the raw reads of each falcon genome. Darker colors indicate fewer distinct 27-mers and lighter colors indicate more distinct 27-mers.

Supplementary Figure 2: counts of sites that are heterozygous for small variants in each genome using three methods of small variant calling: 1) alignment of Supernova assemblies to a pseudohaplotype of the kestrel genome with MUMmer; 2) Alignment of raw reads to a pseudohaplotype of the same genome with Longranger; 3) alignment of Supernova assemblies to a pseodohaplotype of the same genome with MUMmer.

Supplementary Figure 3: Distributions of GC content across 100KB windows for the eight falcon species.

Supplementary Figure 4: Distributions of GC content across 1MB windows for the eight falcon species.

Supplementary Figure 5: PSMC results of demographic trends for each falcon genome time-scaled with a mutation rate based on the collard fly-catcher and recombination rates based on the zebra finch. Time is indicated on the X-axis and is scaled in years by current generation time of each species. Generation times for each species were estimated from published literature. Effective population size is indicated on the Y-Axis in a log-10 scale. Boot straps were performed in 100 replicates and are shown in grey lines.

Supplementary Figure 6: Biases in unique small variants relative to parsimonious ancestral state as: A) Relative abundance of unique small variants, <50 BP (SNVs and Indels), mutations within each genome with Indels, transitions, and transversions separated by color patterns. B) Odds ratios of mutations of mutations from GC to AT with (w/CpG) and without (n/CpG) CpG sites included across all genomes and separated by whether unique mutations are fixed or heterozygous. The critical odds-ratio of 1.0 is indicated by red dashed line.

Supplementary Figure 7: Net unique inserted or deleted base pairs from small indels (<50BP) across 100KB windows in each genome. Ancestral state was determined by parsimony. Boxplots show mean with a central black line with 1st and 3rd quartiles indicated by lower and upper bounds of the box, respectively. Lines extend to up 1.5 times the interquartile range from each quartile. Outliers are removed. A net change of 0 is indicated by a dashed grey line.

Supplementary Figure 8: pairwise differences in means of $\log_2$ (x+1) transformed percent GC content for 100KB windows based on current and ancestral chromosome state with 95% confidence intervals. Differences are corrected for false-discovery rate using Tukey's Honest Significant Differences with a 2-way ANOVA that included assemblies as a blocking effect.

Supplementary Figure 9: pairwise differences in means of $\log_2$ (x+1) transformed residuals of percent CpG content for 100KB windows after regressing against percent GC content based on current and ancestral chromosome state with 95% confidence intervals. Differences are corrected for false-discovery rate using Tukey's Honest Significant Differences with a 2-way ANOVA that included assemblies as a blocking effect.

Supplementary Figure 10: pairwise differences in means of $\log_2$ (x+1) transformed percent CpG content for 100KB windows based on current and ancestral chromosome state with 95% confidence intervals. Differences are corrected for false-discovery rate using Tukey's Honest Significant Differences with a 2-way ANOVA that included assemblies as a blocking effect.

Supplementary Figure 11: pairwise differences in means of $\log_2(x+1)$ transformed odds-ratios of GC-to-AT versus AT-to-GC mutation for 100KB windows based on current and ancestral chromosome state with 95% confidence intervals. Differences are corrected for false-discovery rate using Tukey's Honest Significant Differences with a 2-way ANOVA that included assemblies as a blocking effect.

Supplementary Figure 12: pairwise differences in means of net inserted or deleted bases relative to parsimonious ancestral states for 100KB windows based on current and ancestral chromosome state with 95% confidence intervals. Differences are corrected for false-discovery rate using Tukey's Honest Significant Differences with a 2-way ANOVA that included assemblies as a blocking effect.

Supplementary Figure 13: pairwise differences in means of $\log_2(x+1)$ transformed repetitive bases by current and ancestral chromosome state with 95% confidence intervals. Differences are corrected for false-discovery rate using Tukey's Honest Significant Differences with a 2-way ANOVA that included assemblies as a blocking effect.

Supplementary Figure 14: pairwise differences in means of $\log_2(x+1)$ transformed repetitive bases by current and ancestral chromosome state with 95% confidence intervals after regressing out the effects of GC content. Differences are corrected for false-discovery rate using Tukey's Honest Significant Differences with a 2-way ANOVA that included assemblies as a blocking effect.

1

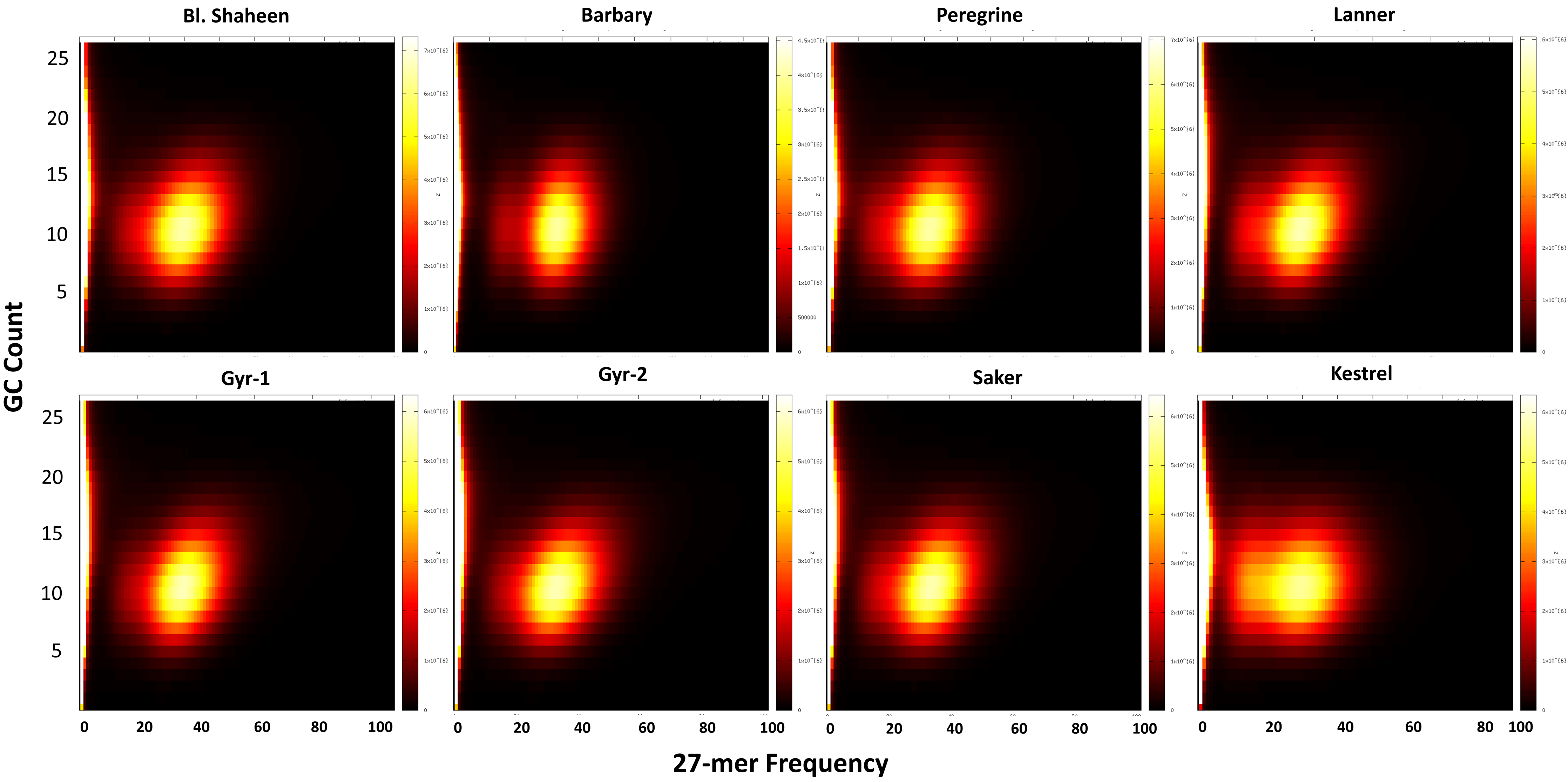
Bl. Shaheen
Barbary
Peregrine
Lanner
Gyr-1
Gyr-2
Saker
Kestrel
GC Count
27-mer Frequency
25
20
15
10
5
0
20
40
60
80
100

2

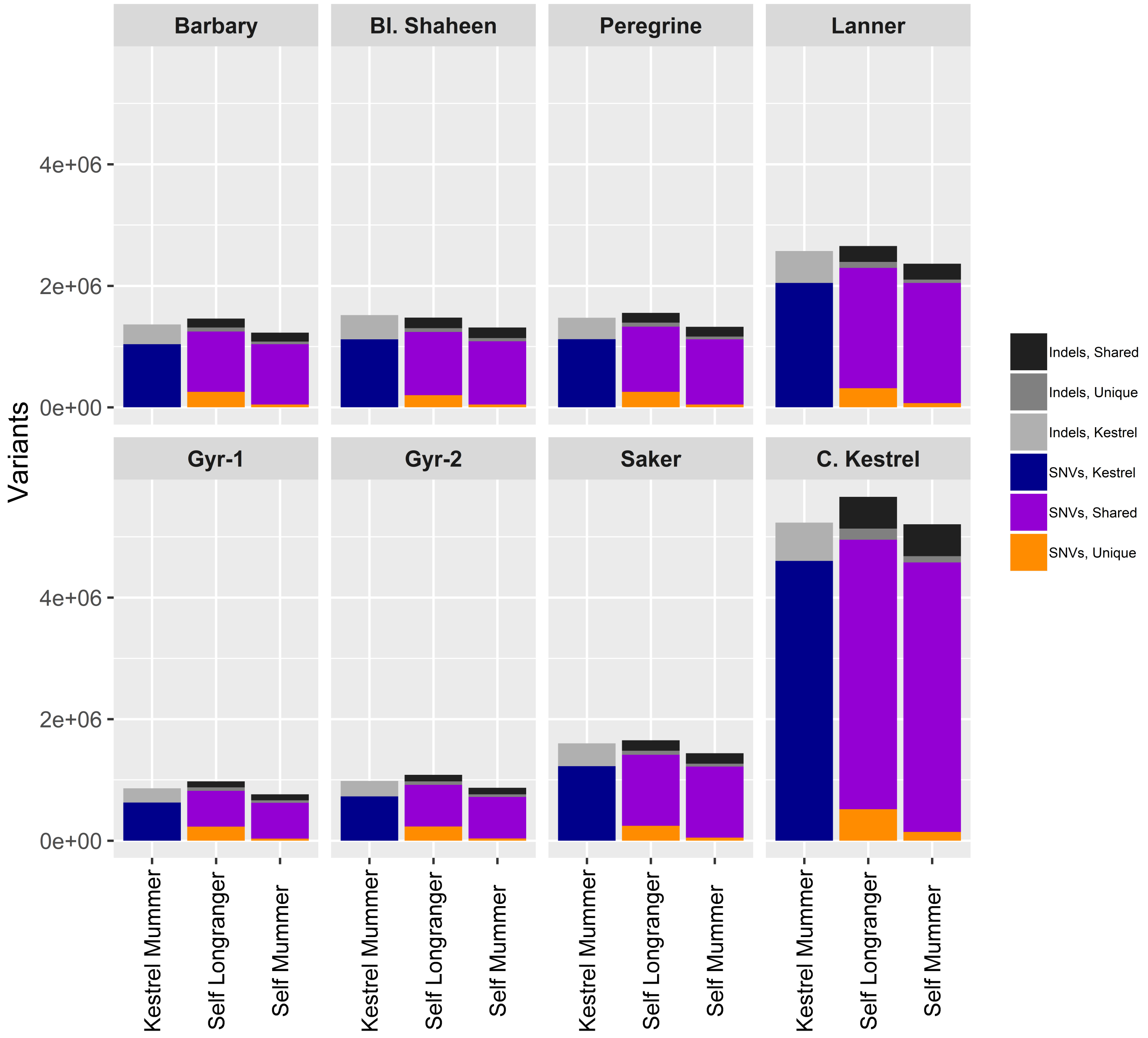
Barbary
Bl. Shaheen
Peregrine
Lanner
Gyr-1
Gyr-2
Saker
C. Kestrel
Variants
4e+06
2e+06
0e+00
Kestrel Mummer
Self Longranger
Self Mummer
Indels, Shared
Indels, Unique
Indels, Kestrel
SNVs, Kestrel
SNVs, Shared
SNVs, Unique

3

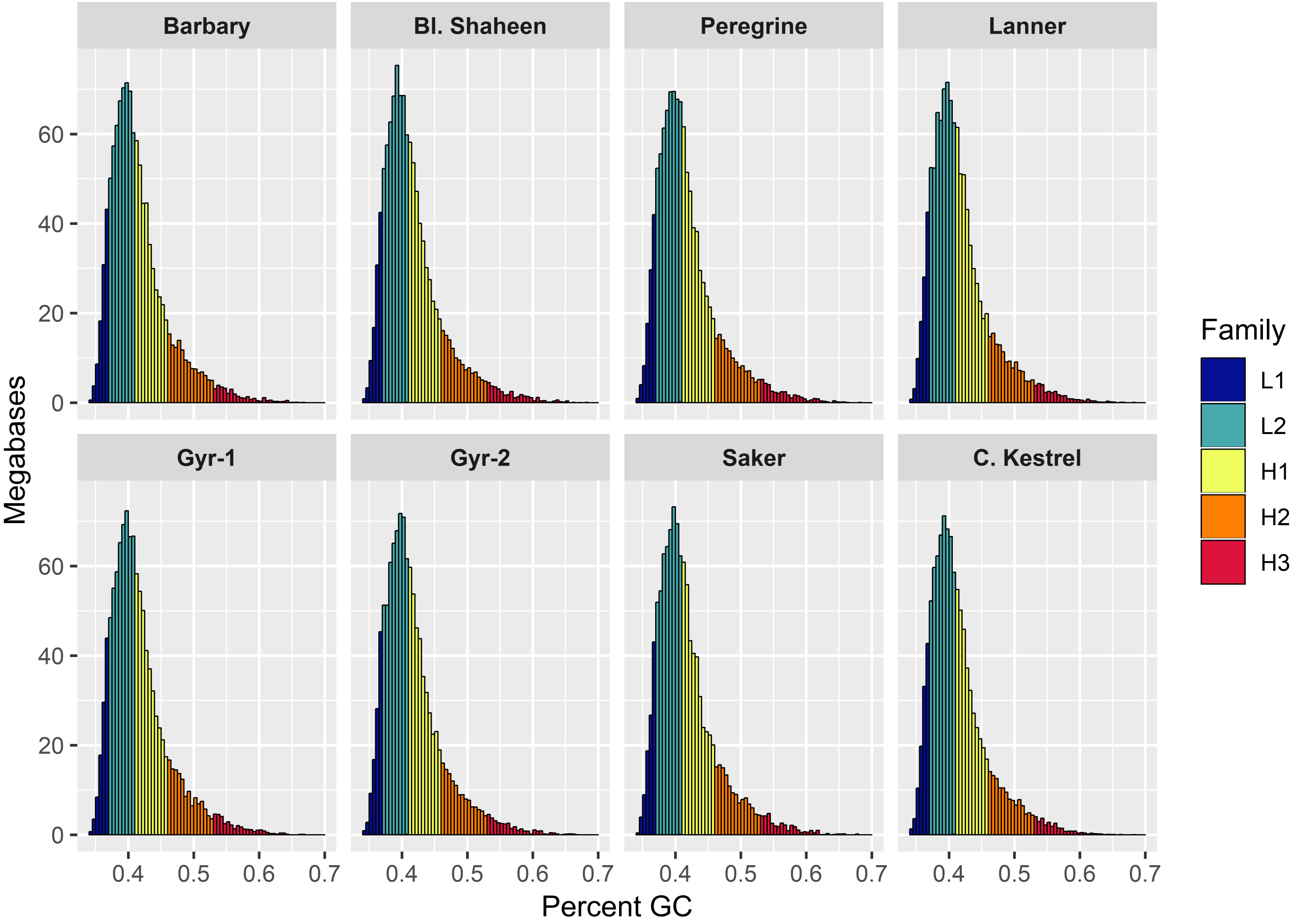
Barbary
Bl. Shaheen
Peregrine
Lanner
Gyr-1
Gyr-2
Saker
C. Kestrel
Megabases
0
20
40
60
0.4
0.5
0.6
0.7
Percent GC
Family
L1
L2
H1
H2
H3

4

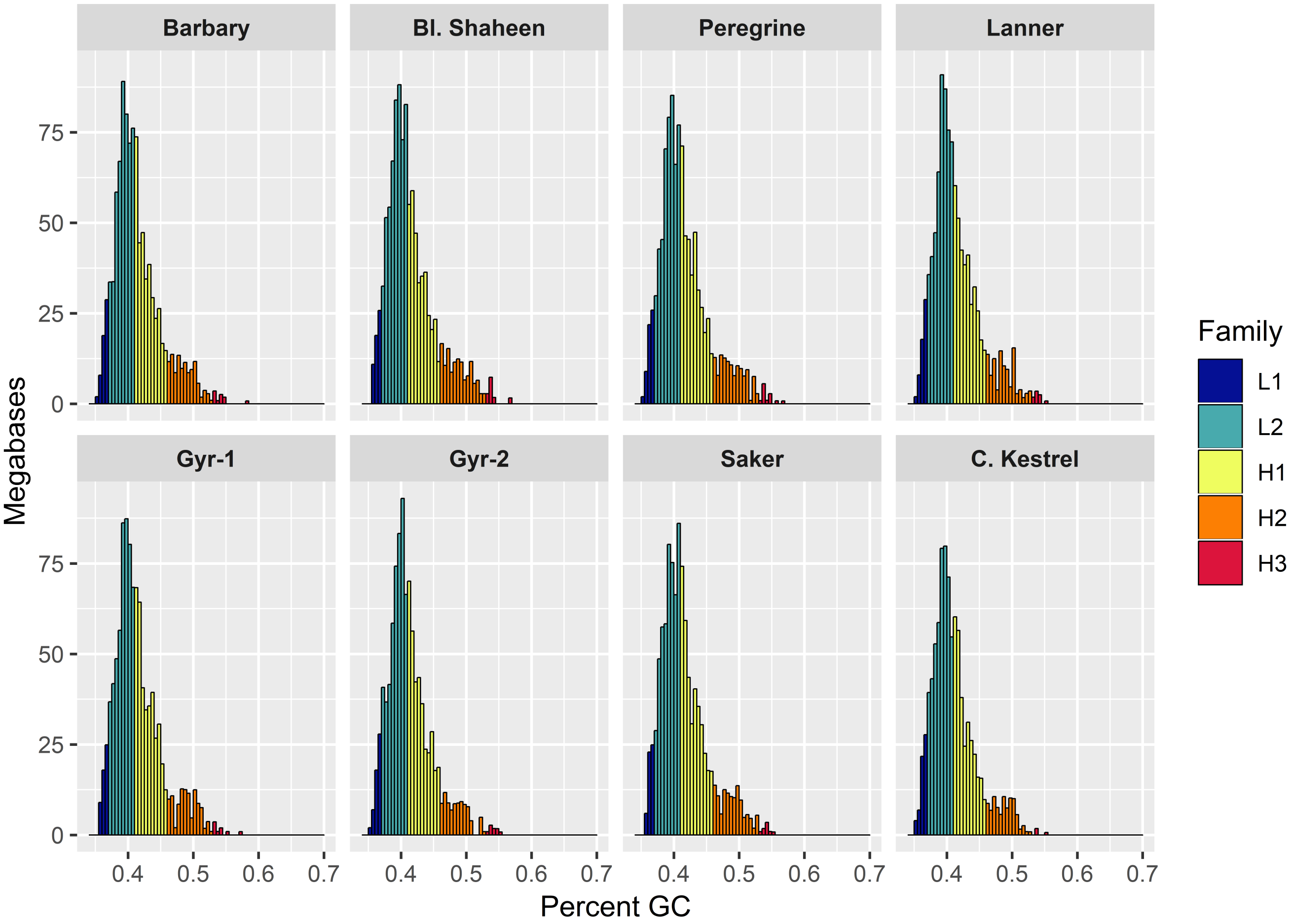
Barbary
Bl. Shaheen
Peregrine
Lanner
Gyr-1
Gyr-2
Saker
C. Kestrel
Megabases
75
50
25
0
0.4
0.5
0.6
0.7
Percent GC
Family
L1
L2
H1
H2
H3

5

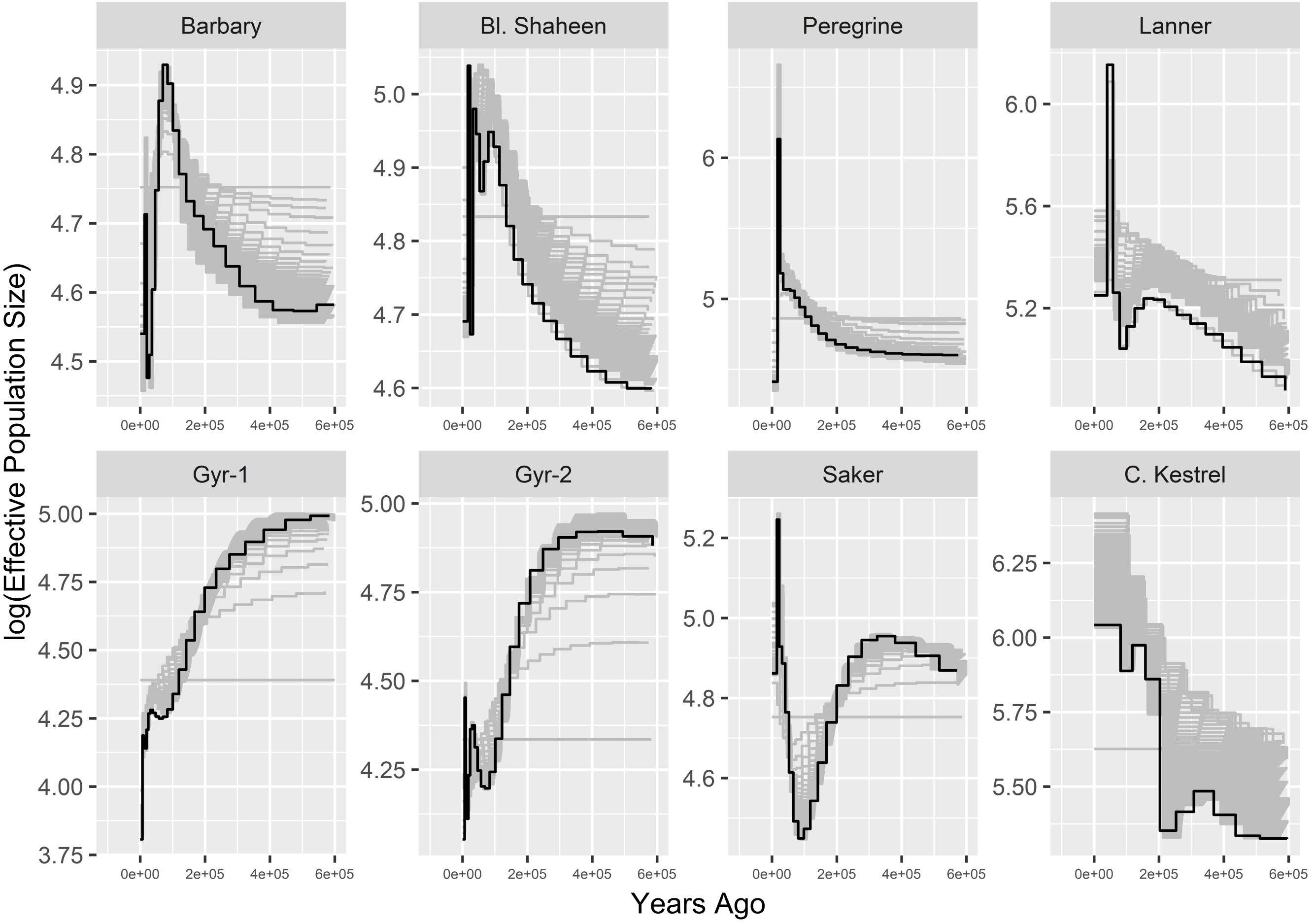

Barbary
Bl. Shaheen
Peregrine
Lanner
Gyr-1
Gyr-2
Saker
C. Kestrel
4.9
4.8
4.7
4.6
4.5
5.0
4.9
4.8
4.7
4.6
6
5
6.0
5.6
5.2
5.00
4.75
4.50
4.25
4.00
3.75
5.00
4.75
4.50
4.25
5.2
5.0
4.8
4.6
6.25
6.00
5.75
5.50
0e+00
2e+05
4e+05
6e+05
log(Effective Population Size)
Years Ago

6

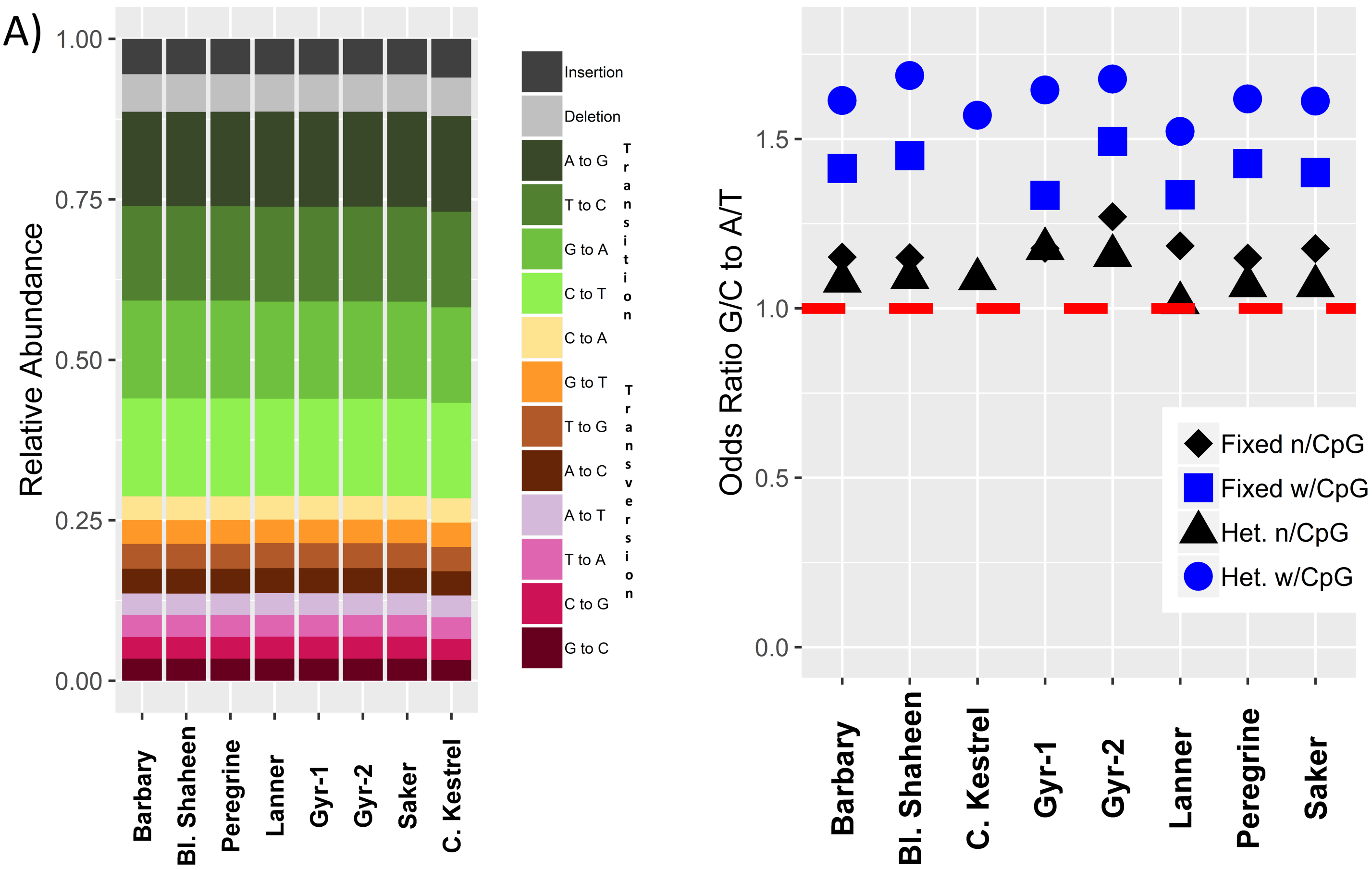
A)
Relative Abundance
1.00
0.75
0.50
0.25
0.00
Barbary
Bl. Shaheen
Peregrine
Lanner
Gyr-1
Gyr-2
Saker
C. Kestrel
Insertion
Deletion
A to G
T to C
G to A
C to T
Transition
C to A
G to T
T to G
A to C
A to T
T to A
C to G
G to C
Transversion
Odds Ratio G/C to A/T
1.5
1.0
0.5
0.0
Fixed n/CpG
Fixed w/CpG
Het. n/CpG
Het. w/CpG
Barbary
Bl. Shaheen
C. Kestrel
Gyr-1
Gyr-2
Lanner
Peregrine
Saker

7

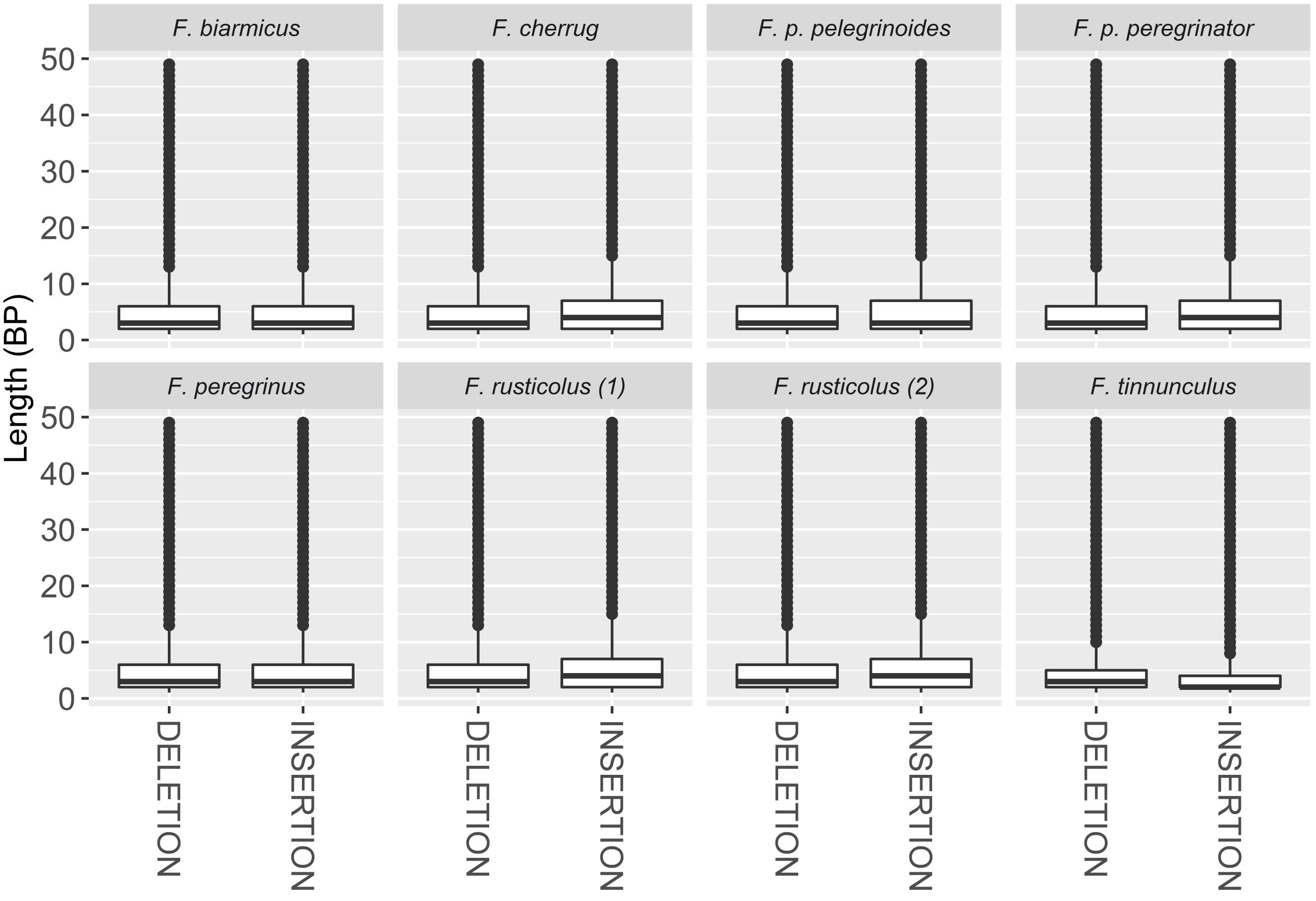
F. biarmicus
F. cherrug
F. p. pelegrinoides
F. p. peregrinator
F. peregrinus
F. rusticolus (1)
F. rusticolus (2)
F. tinnunculus
Length (BP)
0
10
20
30
40
50
DELETION
INSERTION

8

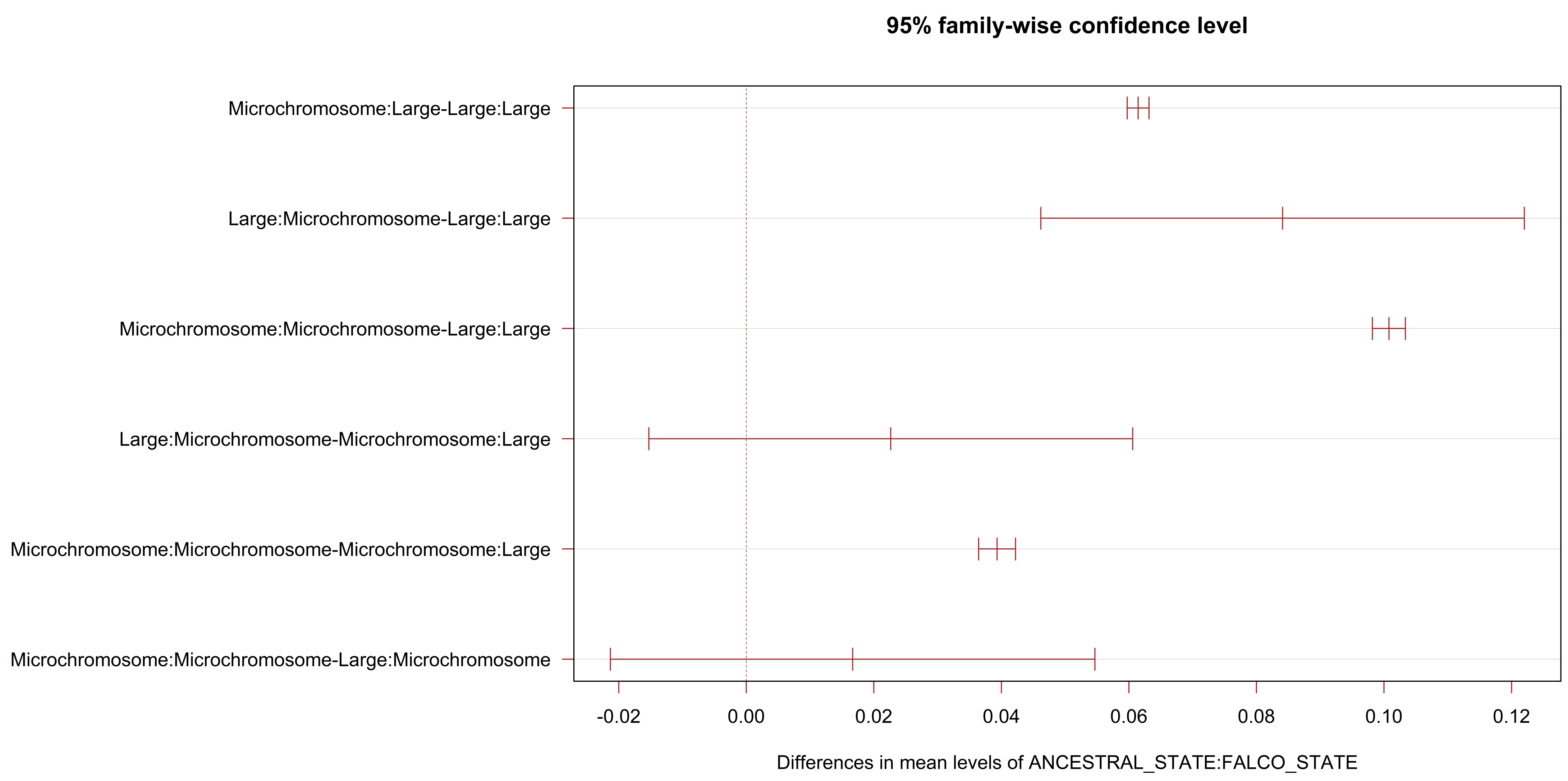

95% family-wise confidence level
Microchromosome:Large-Large:Large
Large:Microchromosome-Large:Large
Microchromosome:Microchromosome-Large:Large
Large:Microchromosome-Microchromosome:Large
Microchromosome:Microchromosome-Microchromosome:Large
Microchromosome:Microchromosome-Large:Microchromosome
-0.02
0.00
0.02
0.04
0.06
0.08
0.10
0.12
Differences in mean levels of ANCESTRAL_STATE:FALCO_STATE

9

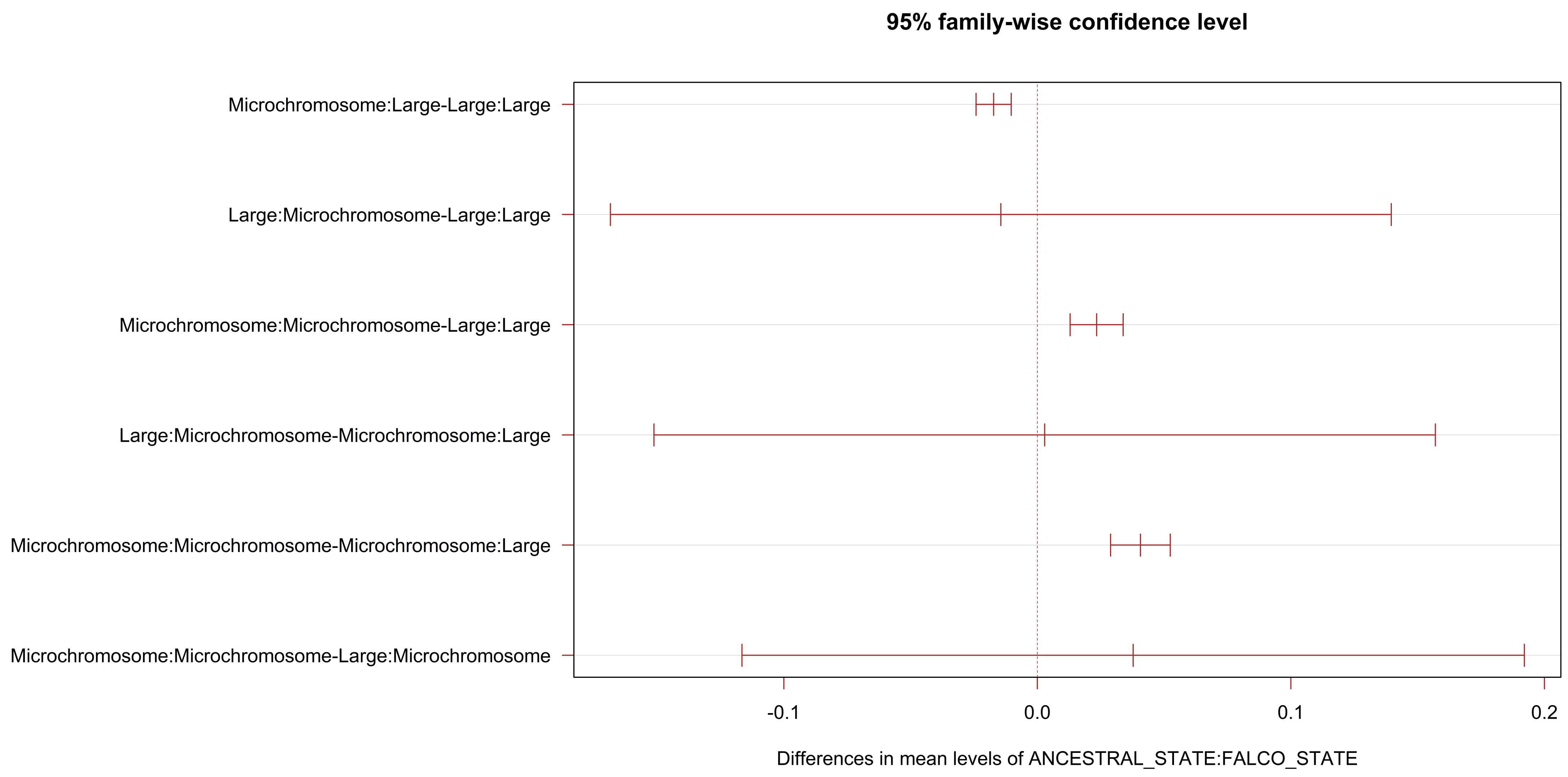
95% family-wise confidence level
Microchromosome:Large-Large:Large
Large:Microchromosome-Large:Large
Microchromosome:Microchromosome-Large:Large
Large:Microchromosome-Microchromosome:Large
Microchromosome:Microchromosome-Microchromosome:Large
Microchromosome:Microchromosome-Large:Microchromosome
-0.1
0.0
0.1
0.2
Differences in mean levels of ANCESTRAL_STATE:FALCO_STATE

10

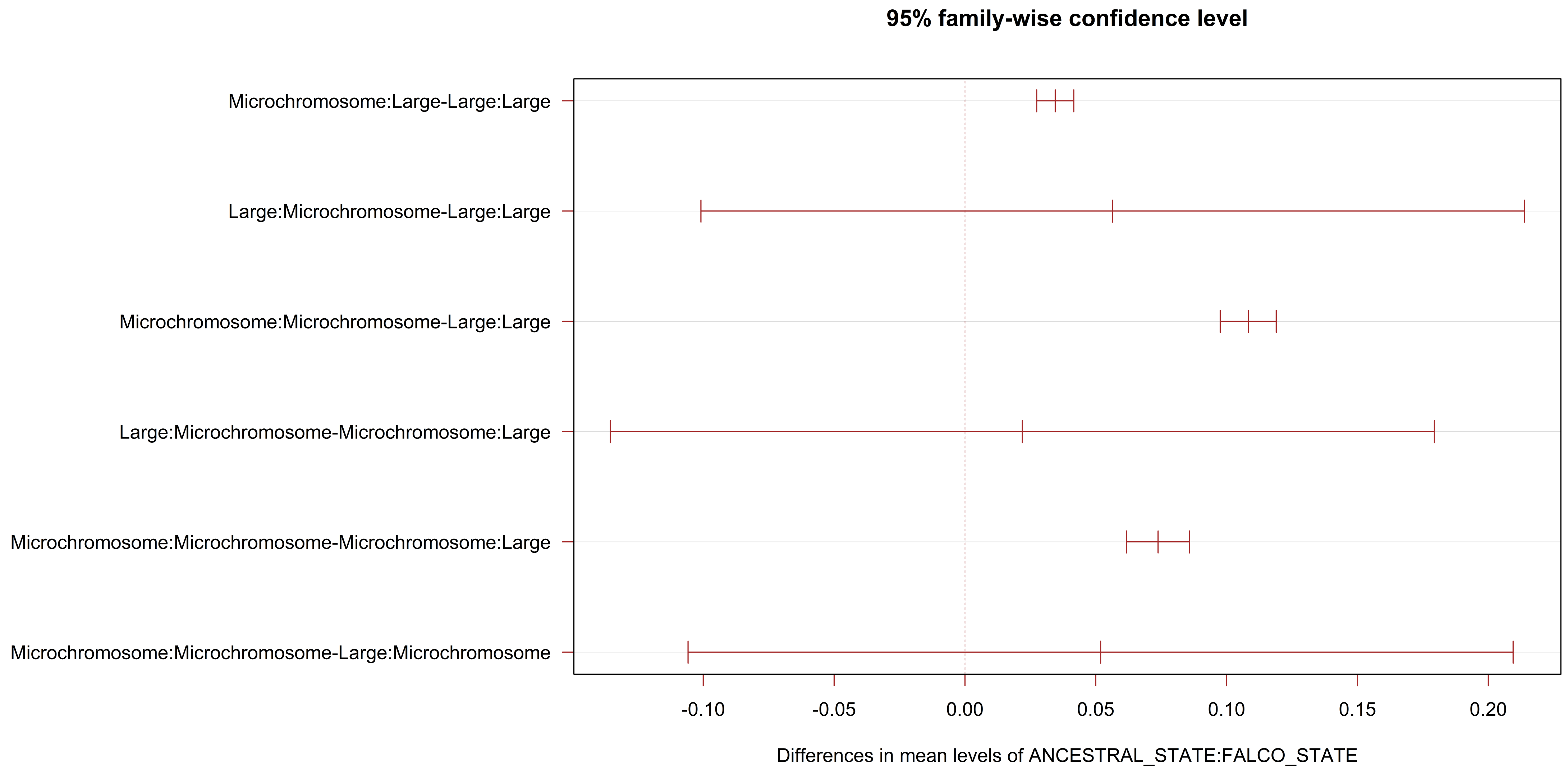
95% family-wise confidence level
Microchromosome:Large-Large:Large
Large:Microchromosome-Large:Large
Microchromosome:Microchromosome-Large:Large
Large:Microchromosome-Microchromosome:Large
Microchromosome:Microchromosome-Microchromosome:Large
Microchromosome:Microchromosome-Large:Microchromosome
-0.10
-0.05
0.00
0.05
0.10
0.15
0.20
Differences in mean levels of ANCESTRAL_STATE:FALCO_STATE

11

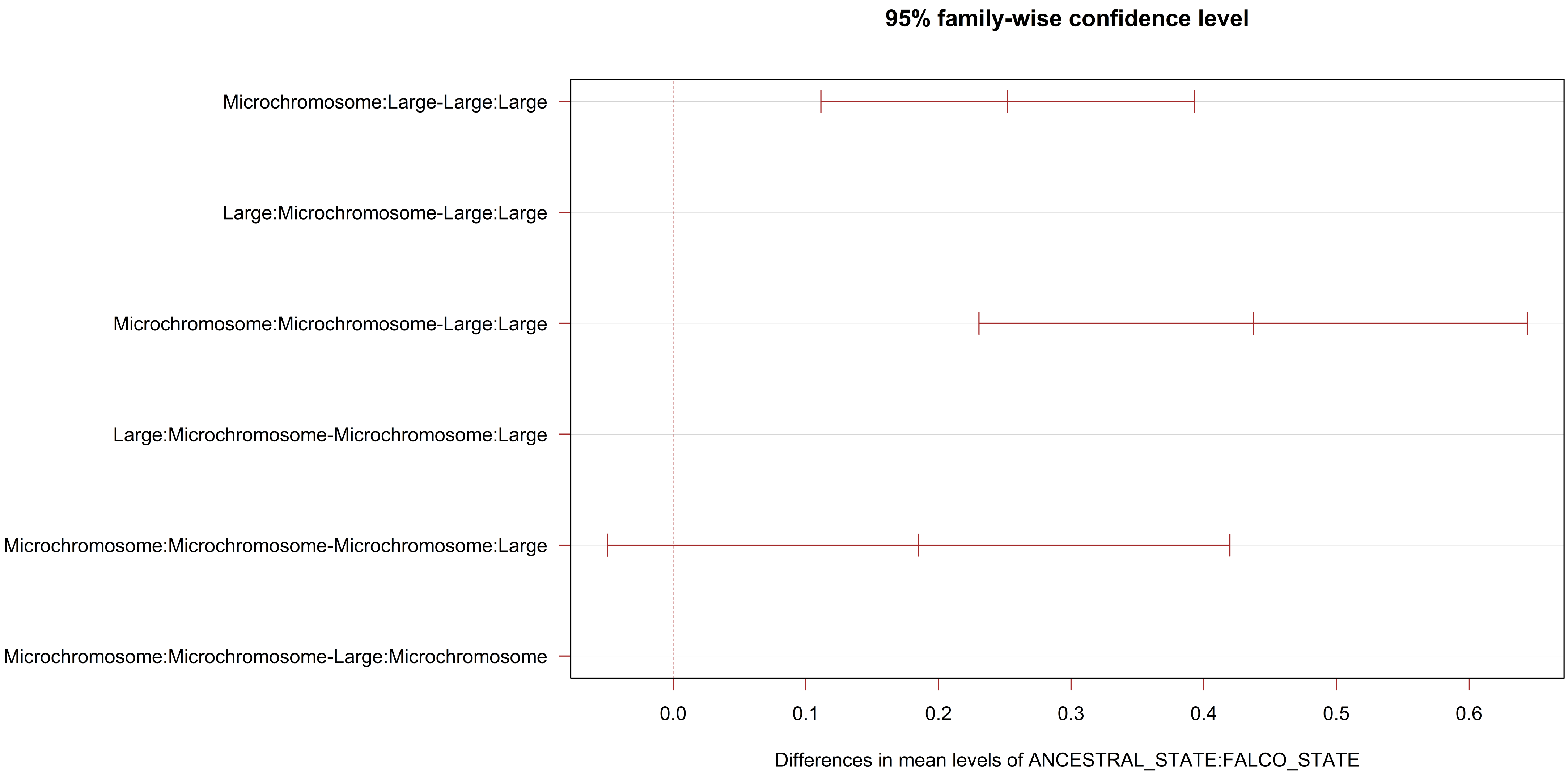
95% family-wise confidence level
Microchromosome:Large-Large:Large
Large:Microchromosome-Large:Large
Microchromosome:Microchromosome-Large:Large
Large:Microchromosome-Microchromosome:Large
Microchromosome:Microchromosome-Microchromosome:Large
Microchromosome:Microchromosome-Large:Microchromosome
0.0
0.1
0.2
0.3
0.4
0.5
0.6
Differences in mean levels of ANCESTRAL_STATE:FALCO_STATE

12

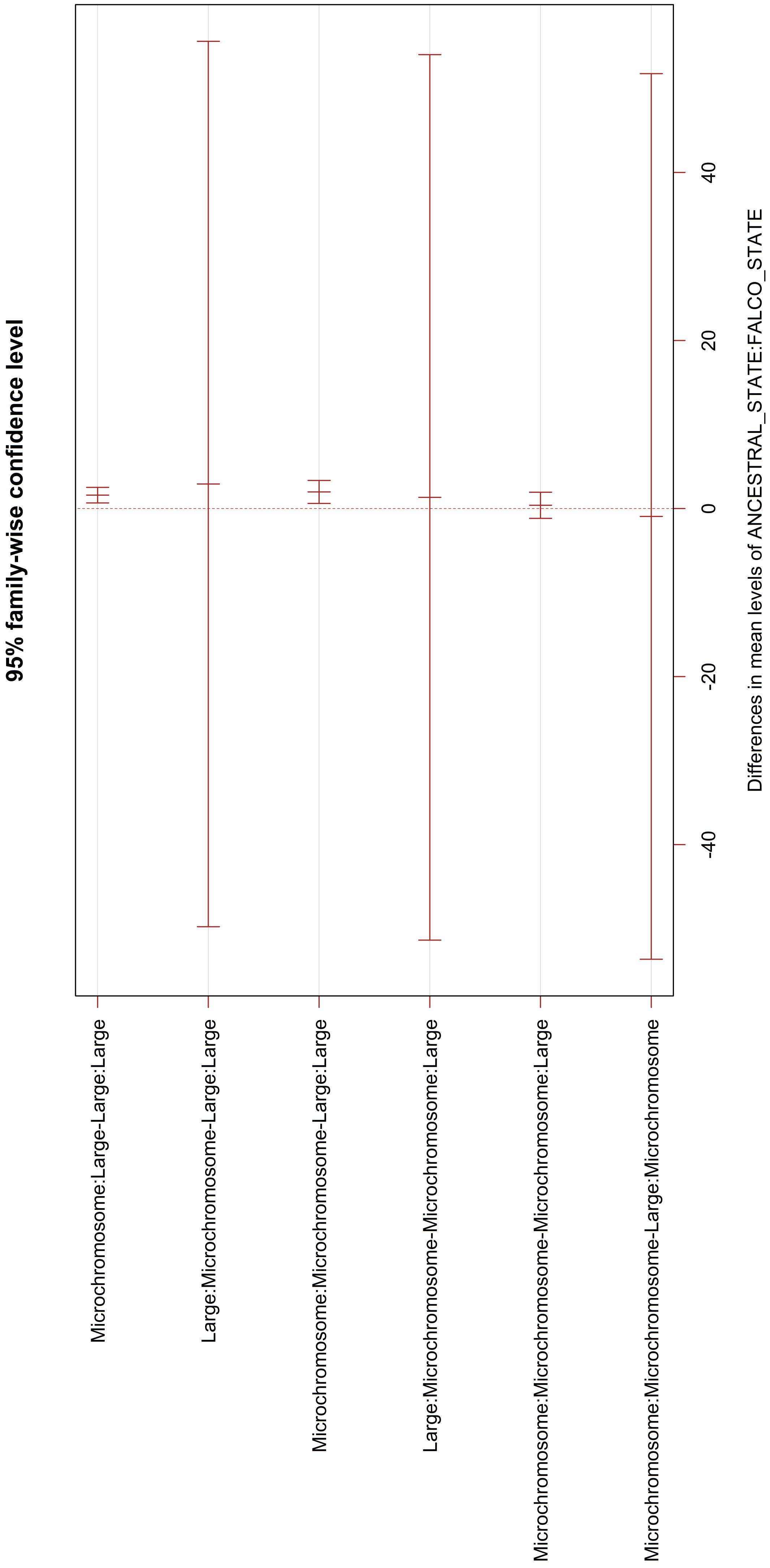

95% family-wise confidence level
Microchromosome:Large-Large:Large
Large:Microchromosome-Large:Large
Microchromosome:Microchromosome-Large:Large
Large:Microchromosome-Microchromosome:Large
Microchromosome:Microchromosome-Microchromosome:Large
Microchromosome:Microchromosome-Large:Microchromosome
-40
-20
0
20
40
Differences in mean levels of ANCESTRAL_STATE:FALCO_STATE

13

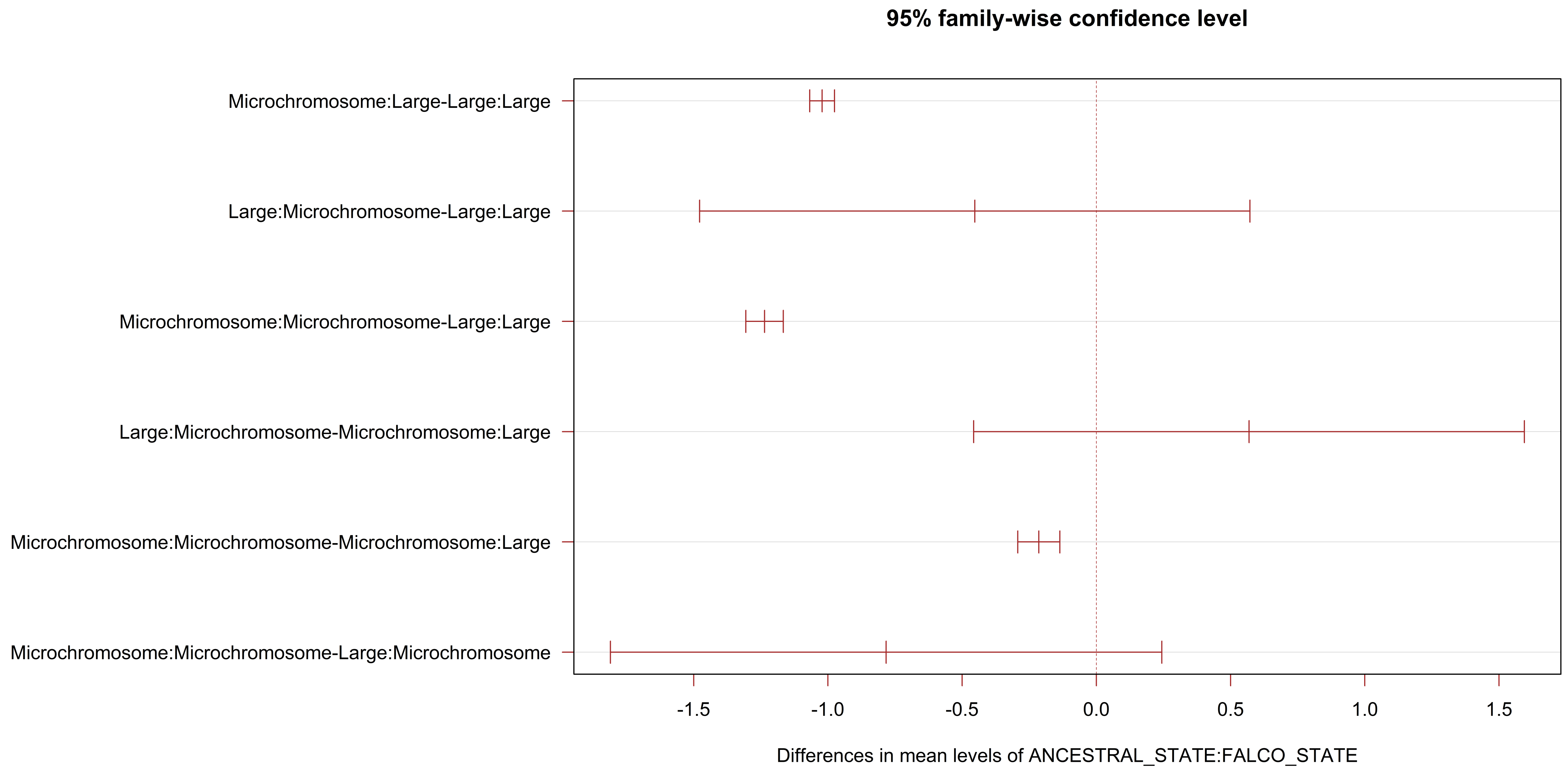
95% family-wise confidence level
Microchromosome:Large-Large:Large
Large:Microchromosome-Large:Large
Microchromosome:Microchromosome-Large:Large
Large:Microchromosome-Microchromosome:Large
Microchromosome:Microchromosome-Microchromosome:Large
Microchromosome:Microchromosome-Large:Microchromosome
-1.5
-1.0
-0.5
0.0
0.5
1.0
1.5
Differences in mean levels of ANCESTRAL_STATE:FALCO_STATE

14

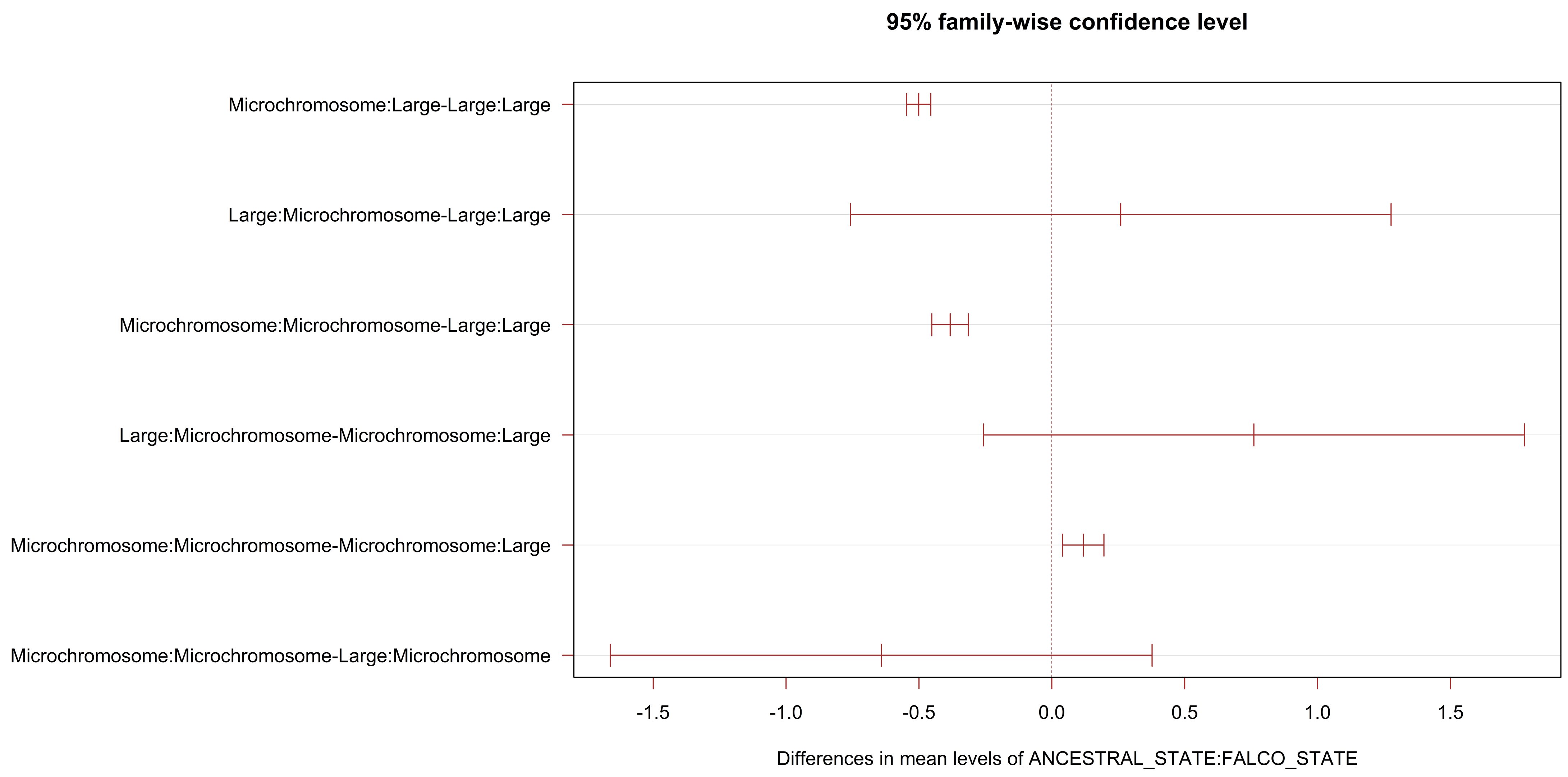

95% family-wise confidence level
Microchromosome:Large-Large:Large
Large:Microchromosome-Large:Large
Microchromosome:Microchromosome-Large:Large
Large:Microchromosome-Microchromosome:Large
Microchromosome:Microchromosome-Microchromosome:Large
Microchromosome:Microchromosome-Large:Microchromosome
-1.5
-1.0
-0.5
0.0
0.5
1.0
1.5
Differences in mean levels of ANCESTRAL_STATE:FALCO_STATE
