# Supplementary material for: Linked-Read Sequencing of Eight Falcons Reveals a Unique Genomic Architecture in Flux
Source: Genome Biol Evol. 2022 Jun 14;14(6):evac090. doi: 10.1093/gbe/evac090 (PMC9214253; doi:10.1093/gbe/evac090)
Supplement: evac090_Supplementary_Data [file evac090_supplementary_data.zip › Supplementary tables.pdf]

	Supernova		Longranger	
**Genome**	**%Phased**	**Phaseblock N50 (MB)**	**% Phased**	**Phaseblock N50 (MB)**
Lanner	63.57	12.11	99.7099	9.84557
Saker	55.24	6.71	99.7242	7.491453
Peregrine	51.52	6.24	99.6727	6.911943
Barbary	53.38	3.31	99.6297	5.298326
Bl. Shaheen	55.93	13.9	99.7373	10.48784
Gyr-1	49.95	1.04	99.5161	2.411638
Gyr-2	49.21	1.27	99.6567	2.744265
C. Kestrel	57.19	7.49	99.5025	4.52376

Supplementary Table 1: Phasing Information

Genome	Hetdist	Het. SNVs	P-Het. SNV	Unique SNVs	N50	P-N50	BUSCOs	P-BUSCOS
Lanner	0.576	2,294,326	-	1,753,304	28.94	-	94.4%	-
Saker	0.961	1,413,295	761,748	707,795	29	4.15[1]	95.1%	-
Peregrine	1.04	1,247,935	879,812	991,747	25.88	3.89[1]/26.78[4]	94.8%	-
Barbary	1.11	1,240,120	-	815,803	26.06	-	94.3%	-
Bl. Shaheen	1.05	1,328,280	-	779,477	40.62	-	93.8%	-
Gyr-1	1.75	820,996	-	415,270	20.6	91.09[2]	95.1%	-
Gyr-2	1.52	917,480	-	414,658	17.12	91.09[2]	95.2%	-
C. Kestrel	0.250	4,952,485	5.152,212	2,302,783*	10.58	21.23	91.1%	93.90[3]

Supplementary Table 2: Reference Genome Comparisons

General information on assemblies with comparisons to previously published ("P-") genomes where possible: [1]Zhan *et al.*, 2013; [2]Vertebrate Genomes Project; [3]Cho *et al.*, 2019; [4]Reference-Assisted Assembly with BACA and FISH assistance from Royal Veterinary College based on previous peregrine assembly

Het. SNVs=Number of heterozygous SNVs called by Longranger

P-Het.SNVs=Number of heterozygous SNVs called in previously published genomes

Unique SNVs=diploid counts of SNVs relative to Kestrel MUMmer alignment that are unique to genome

Genome	Total	Unaligned	Shared	Unique	Heterozygous
Lanner	103(105,585)	0	66(53,509)	37(56,076)	41(19,461)
Barbary	112(150,107)	0	75(83,296)	37(66,811)	26(12,093)
Peregrine	117(113,413)	14(30,140)	82(90,759)	21(12,514)	21(5,512)
Saker	119(138,130)	4(1,670)	87(105,162)	29(31,298)	45(48,178)
Gyr-1	106(104,188)	4(1,952)	74(71,409)	28(30,827)	14(2,878)
Gyr-2	110(123,288)	4(1,948)	61(40,867)	45(80,473)	20(25,552)
Bl. Shaheen	107(127,224)	0	78(84,424)	29(42,800)	19(8,741)
Kestrel	116(110,209)	4(1,678)	24(56,991)	88(51,540)	64(26,756)

Table 3: Diploid NUMTs Counts (Base Pairs) within Each Genome

Total: all diploid NUMTs

Unaligned: NUMTs unaligned to reference (kestrel haplotype)

Shared: NUMTs with shared synteny in other genomes

Unique: NUMTs without shared synteny in other genomes

Heterozygous: NUMTs that only occur on one haplotype of the diploid genome

Genome	ChiSquare	Odds_Ratio	P_Value	Equilibrium Fixed	Equilibrium Het.	pGC
Barbary	31576.2	1.535857	0	41.4410	38.2660	42.04
Bl. Shaheen	33476.58	1.57026	0	40.8065	37.2145	42.02
Gyr-1	16795.41	1.556421	0	42.8520	37.8171	42.27
Gyr-2	16857.06	1.559136	0	40.1186	37.3601	42.25
Lanner	55042.03	1.471315	0	42.8272	39.6525	42.11
Peregrine	37624.09	1.528763	0	41.2030	38.1974	42.05
Saker	27920.76	1.544466	0	41.6546	38.2931	42.14

Supplementary Table 4: Genomic Equilibrium Calculations with CpG Sites. Chi-Square values, odds ratios (GC to AT bias), and p-values denote deviation from equilibrium of AT→GC and GC→AT. "Equilibrium Fixed" denotes the GC content at which falcon genomes would be in equilibrium based on unique fixed mutations. "Equilibrium Het" denotes the GC content at which falcon genomes would be in equilibrium based on unique heterozygous mutations. pGC denotes the current percent GC content of the assemblies.

Genome	ChiSquare	Odds_Ratio	P_Value	Equilibrium Fixed	Equilibrium Het.	pGC
Barbary	695.2359	1.088748	3.25E-153	46.47704	48.0733	42.04
Bl. Shaheen	1042.97	1.110919	8.21E-229	46.51452	47.8217	42.02
Gyr-1	878.0893	1.144337	5.69E-193	45.92803	45.9573	42.27
Gyr-2	995.1389	1.153324	2.05E-218	44.04208	46.3874	42.25
Lanner	969.8524	1.070584	6.42E-213	45.77625	49.5933	42.11
Peregrine	967.4478	1.095474	2.14E-212	46.53808	48.3961	42.05
Saker	1270.658	1.128194	2.69E-278	45.94823	48.3916	42.14

Supplementary Table 5: Genomic Equilibrium Calculations without CpG Sites. Chi-Square values, odds ratios (GC to AT bias), and p-values denote deviation from equilibrium of AT→GC and GC→AT. "Equilibrium Fixed" denotes the GC content at which falcon genomes would be in equilibrium based on unique fixed mutations. "Equilibrium Het" denotes the GC content at which falcon genomes would be in equilibrium based on unique heterozygous mutations. pGC denotes the current percent GC content of the assemblies.

Genome	Windows	Falco Macro	Falco Intermediate	Falco Micro	Falco Ambiguous	Falco Unaligned	Ancestral Macro	Ancestral Intermediate	Ancestral Micro	Ancestral Ambiguous	Ancestral Unaligned
Lanner	11569	7029	1341	254	2233	712	2731	234	959	7644	1
Saker	11583	7003	1328	254	2255	743	2701	237	974	7669	2
Peregrine	11591	7063	1345	248	2302	633	2719	234	977	7660	1
Barbary	11548	7016	1352	249	2279	652	2726	232	961	7627	2
Bl. Shaheen	11683	6996	1354	260	2375	698	2769	237	960	7716	1
Gyr-1	11554	6986	1311	244	2284	729	2692	237	956	7669	0
Gyr-2	11481	6925	1318	244	2241	753	2689	231	954	7606	1
C. Kestrel	11331	6904	1310	233	2308	576	2730	226	932	7443	0

Supplementary Table 6: Number of 100KB windows in each genome annotated as belonging to current (Falco…) and ancestral (Ancestral…) chromosome types based on alignment to chromosome-scale *Falco* and other Inopinaves genome assemblies respectively. Microchromosomes are defined as chromosomes of 20MB or less and Macrochromosomes are defind as chromosomes of greater than 40MB with intermediate chromosomes defined as those of intermediate sizes between 20-40 MB. AncestralUnaligned denotes windows that failed to align to any chromosomes in other Inopinaves.
